# Provider Attribution in Medicare: Challenges and Solutions

**DOI:** 10.1111/1475-6773.70062

**Published:** 2025-10-28

**Authors:** Caroline S. Carlin, Roger Feldman, Jeah Jung

**Affiliations:** ^1^ Department of Family Medicine and Community Health University of Minnesota Minneapolis Minnesota USA; ^2^ Division of Health Policy and Management University of Minnesota Minneapolis Minnesota USA; ^3^ Department of Health Administration and Policy George Mason University Fairfax Virginia USA

**Keywords:** Medicare Advantage, National Provider Identifiers, retrospective provider attribution, Traditional Medicare

## Abstract

**Objective:**

To enhance National Provider Identifier (NPI) and specialty information available in Medicare Advantage (MA) encounter data and use the enhanced data to evaluate methods for retrospective attribution of the patient's usual clinician, comparing results across MA and Traditional Medicare (TM) populations.

**Study Setting and Design:**

We fill in missing clinician identifiers and specialty codes in MA encounter data using Centers for Medicare and Medicaid Services (CMS) and publicly available provider datasets. We attributed patients to the usual clinician using 16 methodological options, comparing the performance of these attribution methods in MA and TM.

**Data Sources and Analytic Sample:**

We used a 20% sample of MA encounter data and TM claims data for 2016–2022, incorporating information from CMS's Medicare Data on Provider Practice and Specialty, archived data from the National Plan and Provider Enumeration System, and specialty‐taxonomy crosswalks derived from CMS publications.

**Principal Findings:**

For MA, we identified individual NPIs for 83% of medical claims in 2016, improving to 89% in 2022. Among MA medical claims billed by physicians and advanced practice providers, 95% of NPIs were for individual clinicians by 2022. In total, we identified individual or organization NPIs and specialty codes for over 99% of medical encounters in both TM and MA in all years. Rates of patient attribution were stable over time, and most methods had similar performance in MA and TM. We recommend a hierarchical attribution method that resulted in the highest fraction attributed with good consistency of attributed clinician year over year. Published reference files and SAS code make these NPI identification and patient attribution methods accessible.

**Conclusions:**

Our methods allow researchers to identify provider NPIs that can be matched to external clinician data, used to attribute patients to a usual source of care, or to fit clinician fixed effects in studies of MA and TM.

**Summary**

- What is known on this topic
  ○ Retrospective attribution of patients to providers is an important task supporting financial incentive programs, care management, and research activities.
  ○ Medicare Advantage (MA) encounter data are often incomplete, and missing provider information is common, making imputation of usual source of care difficult.
- What this study adds
  ○ We identify data sources and processes to identify National Provider Identifiers and specialty codes in MA encounter data at rates comparable to those in Traditional Medicare (TM) claims.
  ○ We use this enhanced provider data to attribute patients to their usual source of care, evaluating the performance of MA and TM attribution across 16 methodological choices, including robustness checks.
  ○ We provide sample SAS code and reference files, making these proposed methods accessible to a wide variety of users and relevant to settings beyond Medicare.

## 1 Introduction

Medicare is the federal health insurance program for older adults and some disabled people in the U.S., with government spending reaching $1.0 trillion in 2023 [1]. Medicare Advantage (MA) is a private alternative to Traditional Medicare (TM) coverage. MA is growing rapidly, from 25% of Medicare beneficiaries in 2010 to 54% in 2024 [2]; the Congressional Budget Office projects nearly two‐thirds of Medicare beneficiaries will be in MA by 2034 [3]. Because of MA's rapidly growing importance, its performance relative to TM has been the subject of a burgeoning literature [4, 5]. This literature was supported when the Centers for Medicare and Medicaid Services (CMS) released MA encounter data similarly structured to TM claims data, beginning with 2015 dates of service.

The ability to link patients to their usual sources of care is an important task in evaluating provider performance. Payers may determine the patient's usual source of care to impose financial responsibility on providers for an episode of care, for management of a specific condition, or for the total cost of care for all conditions. Payers may identify the patient's usual clinician to reward quality of care or to penalize poor quality. Researchers may attribute patients to their usual clinician to evaluate care processes and determine factors that lead to efficient care delivery, to incorporate external data sources linked by clinician identifiers, or to facilitate analytic techniques such as clinician fixed effects.

Despite the growing importance of MA, studies examining associations between the characteristics of an MA patient's usual clinician and the patient's care delivery are sparse [6]. This gap exists primarily because the ability to attribute patients to clinicians in MA encounter data is limited by missing clinician information [7]. The lack of clinician information is one challenge among many in using MA encounter data.

This study has two aims. First, we identify data sources and processes to enhance clinician information available in the MA data. Second, using this enhanced clinician data, we evaluate the performance of methods to attribute patients to a usual source of care [8, 9, 10, 11].

### 1.1 Challenges in Using Medicare Advantage Encounter Data

Incomplete submission of patient encounters has been a concern since CMS originally released the 2015 data. While the quality of the data is improving, gaps still exist [7]. Jung et al. [12] compared encounter data to external sources and found that 48% of MA contracts submitted reasonably complete data in 2015, improving to 61% by 2018. We applied a similar approach from 2015 to 2022 and found over 90% of contracts had relatively complete data in 2022 (Supporting Information), indicating an increasing probability of observing a complete picture of patient encounters. We test the impact of incomplete data submission on patient attribution to the usual source of care in our Supporting Information.

Another gap in MA encounter data is the lack of financial data. Because of proprietary concerns, MA plans do not submit financial data. Jung's team proposed methods of computing standardized fees based on TM claims and used these to measure resource use in MA plans [12]. This is an important enhancement when provider attribution relies on a measure of resource use, such as allowed charges.

Finally, National Provider Identifier (NPI) data at the clinician level are often missing in MA encounter data. Based on our calculations, 65% of outpatient medical claims in 2016 Carrier and Outpatient Facility files had valid clinician NPIs at the base claim or line level, improving to 76% in 2022. When clinician NPI is missing, the organization NPI can be used (accounting for another 34% in 2016, 24% in 2022). Many organization NPIs represent individual clinicians. Missing clinician specialty information is another important gap in MA encounter data. Some MA clinician NPIs do not match CMS's Medicare Data on Provider Practice and Specialty (MD‐PPAS), so clinician specialty codes cannot be determined.

### 1.2 Attribution in the Context of Medicare Advantage Comparisons With Traditional Medicare

Many research questions involve comparing care delivery in MA versus TM. They need to account for differences in beneficiary characteristics, for example, MA enrollees, on average, are more racially diverse and more likely to have dual Medicare‐Medicaid coverage [13]. In addition to demographic controls, health risk measures are often used in research. Because MA plans' payments are risk adjusted, plans and their contracted providers have an incentive to be more diligent about recording all possible diagnoses for each patient, to maximize payments [14, 15]. Thus bias may be introduced in health risk controls through differences in coding intensity.

Research questions that rely on patient attribution to clinicians or health care organizations (HCOs) have an additional potential source of bias when missingness of provider descriptors in the MA encounter data is nonrandom. Nonrandom missingness would cause systematic differences between MA and TM patterns of provider attribution. Thus, obtaining provider information in MA encounter data that is comparable to TM provider data is a key defense against increasing bias due to differences in provider attribution.

### 1.3 The Process of Patient‐Provider Attribution

Payers' claims data have become a common source of information for retrospectively attributing patients to their providers. The primary care physician (PCP) has notable advantages over other members of the patient's care team in coordinating the patient's care across settings [16, 17]. The PCP is arguably in the best position to facilitate exchanges regarding the patient's care needs between specialists and across care settings. For this reason, provider attribution often focuses on finding the patient's usual source of primary care. For example, the Medicare Shared Savings Program's algorithm first attributes the patient to the HCO with a plurality of primary care encounters, then uses specialty encounters when no primary care encounters occurred [18].

#### 1.3.1 Attribution Level

The level of provider attribution can be the patient's HCO, their practice location, their medical team within a location, or an individual clinician. The level of attribution depends on the reason for attribution. For example, financial responsibility for an episode of care is often tied to the specialist managing the episode, who may be the clinician completing a surgical procedure [19]. Financial responsibility for primary care or chronic care management can be tied to the clinician or practice location by examining patterns in the patient's primary care encounters. Total cost of care may be managed at the HCO level, as in the Medicare Shared Savings Program [18]. Attribution for quality‐of‐care measurement may be done at the HCO level if minimum quality standards are required for financial rewards, or at the practice location or physician level when done by an HCO to determine quality bonuses [14, 20].

#### 1.3.2 Scope of Encounters Used

After determining the level of attribution, retrospective provider attribution algorithms must determine the types of encounters used. For example, does the algorithm use all outpatient encounters, or only evaluation and management (E&M) visits? Which types of clinicians will be included? Will encounters with advanced practice providers (physicians' assistants and nurse practitioners) be included [21], or only physician encounters? Are physicians limited to PCPs or should specialists be included? Hierarchical attribution may also be considered, as in the two‐step Medicare Shared Savings Program algorithm described above [18].

#### 1.3.3 Attribution Rule

After defining the eligible encounters, the algorithm must specify the rule for attribution. The most common rule is attribution to the provider with a plurality of encounters, but plurality of resource intensity (typically measured by allowed charges) is an alternative [22, 23]. How does the algorithm handle ties for that plurality?

#### 1.3.4 Span of Time

Finally, the span of time for attribution must be defined. Because patients' provider affiliations can change over time, “recency” of the eligible encounters is an important consideration. However, healthy individuals may have few encounters with health care providers; thus periods longer than the most recent year may be considered.

## 2 Methods

### 2.1 Data Sources

The primary data source was CMS's 20% sample of MA encounter and TM claims data [24] for 2016–2022. We included beneficiaries with continuous MA or TM enrollment during the calendar year, full Part A and Part B coverage, and residence in the 50 states or the District of Columbia (details in the Supporting Information). We used outpatient medical encounters in the Carrier (professional services) or Outpatient Facility files for provider attribution. We used the CMS Hierarchical Condition Category software to develop health risk scores from all Carrier, Outpatient Facility and Inpatient Facility files for subgroup analysis [25], excluding chart review records and health risk assessments to reduce bias due to MA/TM coding intensity differences. Patient characteristics and coverage information were drawn from the Master Beneficiary Summary files, with geographic descriptors appended from the American Community Survey (ZIP code) [26], Area Health Resource Files (County) [27] and Rural–Urban Community Codes (ZIP code) [28] to quantify differences in the MA and TM populations.

To enhance provider data, we used CMS's annual MD‐PPAS file. We also used National Plan and Provider Enumeration System (NPPES) data to identify provider taxonomy codes when these codes were missing. Because these data change each year as providers enter and leave the workforce, we accessed the archive of NPPES data available through the National Bureau of Economic Research [29], downloading yearend snapshots. Taxonomy codes were mapped to CMS specialty codes using a crosswalk derived from data available on the CMS website [30, 31] (see Supporting Information).

### 2.2 Methodology for Identification of Clinician NPI and Specialty Codes

Provider data in the TM files are highly complete, with individual‐level NPIs and CMS specialty codes listed for nearly all performing clinicians in the Carrier line file and attending clinicians in the Outpatient base file. However, the MA Carrier and Outpatient files contain few specialty codes, and NPI data for the treating clinicians are often missing.

We filled missing clinician NPI with organization NPI, which populates for almost all records, and created “assigned NPIs” as follows: (1) For Carrier files, use performing clinician NPI from the line file; if it is missing, use rendering physician NPI on the base file, and when rendering physician NPI is missing, use organization NPI on the base file; and (2) For Outpatient files, use attending physician NPI on the base file, and when it is missing, use organization NPI on the base file.

We used the following process to identify CMS specialty codes for MA claims. (1) When the specialty code is recorded in the data, use this code. (2) When the code is missing, use the assigned NPIs to pull specialty from MD‐PPAS. Note that a successful match suggests the organization NPI field contains an individual clinician NPI. (3) When the assigned NPI is unmatched to MD‐PPAS, map the organization taxonomy code from the Outpatient or Carrier base file to the specialty code using the taxonomy‐specialty crosswalk. (4) Finally, if the organization NPI is used but the taxonomy code is not available, map the organization NPI to its NPPES primary taxonomy code, and then to the specialty code using the taxonomy‐specialty crosswalk.

When a Carrier encounter has beneficiary, procedure code and date of service matched with an Outpatient Facility claim [32], we use the NPI/specialty information present on the Carrier file for that encounter.

After CMS specialty codes were determined, we mapped the codes to the following four designations: primary care physician (PCP), specialty care physician (SCP), advanced practice provider (APP), or others (e.g., chiropractors), as documented in the Supporting Information. We retained only encounters with Healthcare Common Procedure Coding System (HCPCS) codes in the medical range (90281–99607), identifying the subset of medical encounters for evaluation and management services (HCPCS codes 99201–99499). Because MA encounter data can include duplicate claims [12], we consolidated all data to the unique encounter at the NPI‐date level before applying the attribution methodology.

### 2.3 Methodology for Attribution to Usual Source of Care

Our basic methodology attributes the patient to the NPI with whom they have a plurality of encounters, using resource intensity as a tiebreaker. Resource intensity is measured by standardized fees, computed from TM data following methods proposed by Jung et al. [12]. We require a patient to have a minimum of 50% of their medical encounters mapped to clinician NPI and specialty information before they can be attributed.

Data constraints and study context can influence the choice of attribution methodology. Thus, we evaluate all 16 permutations of three methodological choices. First, attribution may be based on (1) all medical encounters [“medical encounters”] or (2) only evaluation and management encounters [“E&M encounters”]. Second, attribution may be based on encounters with (1) all physicians and APPs [“all MDs/APPs”], (2) physicians only [“MDs only”], (3) primary care physicians only [“PCPs only”], or (4) primary care physicians, if possible, otherwise encounters with all physicians and APPs [“PCPs then all”]. Note that primary care is identified by the type of clinician, and not HCPCS codes. Finally, attribution may be based on (1) only the most current year of data [“no lookback”] or (2) looking back to the prior year's data if the current year does not result in attribution [“lookback”].

For each method, we compute the fraction of the population attributed to a clinician (“fraction attributed”) to measure the sensitivity of our methods. For those attributed to a clinician in the current year, we compute the fraction of the population attributed to the same NPI in the prior year (“attribution stability”). Note that changing attribution can come from true changes in patient‐clinician affiliation or from the spurious attribution that is our concern. However, it is a useful *relative* measure of attribution accuracy when comparing methods.

### 2.4 Attribution Robustness Tests

We performed six robustness tests (details are in the Supporting Information): (1) Attribution to the TIN level rather than the NPI level; (2) Attribution first to the TIN level and then the NPI within that TIN; (3) Eliminating the resource intensity tiebreaker; (4) Attribution based on resource intensity with the number of encounters as a tiebreaker; (5) Increasing the 50% minimum provider information necessary for attribution; (6) Including APPs as primary care providers in the “PCPs only” and “PCPs then all” methods.

Because some MA plans submit incomplete encounter data, attribution may perform differently when we have a more complete picture of patient encounters. We compare the results for MA contracts deemed to have complete data submission with those with incomplete data submission [12].

As an example of alternative uses of the provider data, we estimate the fraction attributed to an oncologist for patients with cancer identified by CMS's Chronic Condition Warehouse algorithms [33].

Finally, we assess how the fraction attributed and attribution stability vary across patient health risk quartiles.

### 2.5 Statement of Institutional Review

The Institutional Review Board at the Principal Investigator's institution determined this study was exempt human subjects research with a waiver of informed consent.

## 3 Results

### 3.1 Population Characteristics

Table 1 reports summary statistics for the MA and TM populations. Compared to TM, MA enrollees are more racially diverse and more likely to be dually eligible for Medicaid and to receive the Part D low‐income subsidy.

**TABLE 1:** Patient characteristics in study sample.

	Full sample 2018–2022		
	TM	MA	All
*N*	31,767,887	22,244,131	54,012,018
Age: mean (SD)	72.1 (11.6)	72.8 (9.9)	72.4 (10.9)
Sex (%)			
Male	45.44%	43.79%	44.76%
Female	54.56%	56.21%	55.24%
Race (%)			
White, non‐Hispanic	81.63%	71.90%	77.61%
Black, non‐Hispanic	8.45%	12.31%	10.05%
Hispanic	5.75%	10.71%	7.80%
Asian, non‐Hispanic	2.80%	3.97%	3.29%
Other race	1.37%	1.11%	1.25%
Coverage type (%)			
MA HMO		59.40%	
MA PPO		40.18%	
Any dual Medicare/Medicaid	18.15%	19.02%	18.51%
Low‐income subsidy	19.92%	22.56%	21.01%
Full Part D coverage	68.96%	97.45%	80.69%
Current ESRD eligibility	0.07%	0.03%	0.05%
Health status: mean (SD)			
HCC score	1.1 (1.2)	1.2 (1.1)	1.1 (1.1)
Frailty score	0.2 (0.1)	0.2 (0.1)	0.2 (0.1)
Neighborhood descriptors: mean (SD)			
Percent in ZIP with 4‐year degree	32.7 (17.2)	31.1 (16.1)	32.0 (16.8)
Percent in ZIP speaking English only	86.7 (16.5)	84.7 (19.7)	85.9 (17.9)
Percent in ZIP under the Federal Poverty Limit	12.3 (7.7)	13.0 (7.9)	12.6 (7.8)
Hospital beds/1000 in county	2.9 (2.3)	2.9 (1.9)	2.9 (2.1)
MDs/1000 in county	3.3 (2.5)	3.5 (2.4)	3.4 (2.4)
SNF beds/1000 in county	5.7 (3.3)	5.2 (2.8)	5.5 (3.1)
MA penetration in county	33.6 (13.9)	42.0 (12.3)	37.1 (13.9)
Rural ZIP code (%)	26.32%	17.52%	22.70%

Abbreviations: ESRD, end‐stage renal disease; HCC, hierarchical conditional category; HMO, health maintenance organization; MA, Medicare Advantage; MD, medical doctor; PPO, preferred provider organization; SD, standard deviation; SNF, skilled nursing facility; TM, Traditional Medicare.

Exhibits S3–S18 provide similar summaries for the patients attributed by each of the 16 methods evaluated here. All references to exhibits with “S” enumeration are available in Supporting Information. Changes in patient demographics from the full population to each attributed population are relatively small.

#### 3.1.1 Identification of Clinician NPI and Specialty Codes

**FIGURE 1:**
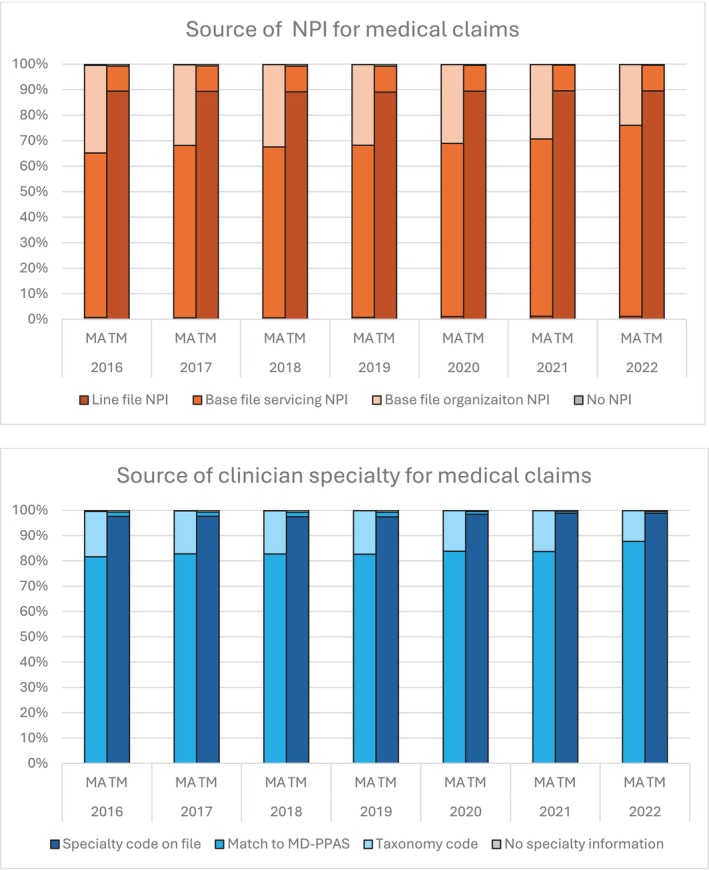
Source of Clinician Information. MA, Medicare Advantage; MD‐PPAS, Medicare Data on Provider Practice and Specialty; NPI, National Provider Identifier; TM, Traditional Medicare.

After applying our hierarchical approach to identify clinician NPI and specialty code, we have similar rates of clinician information completeness between MA and TM encounters, but the sources of data are meaningfully different. The top panel of Figure 1 indicates the source of NPI information for MA and TM medical claims. Essentially all TM NPI information came from clinician NPIs on the line or base files (99.6% in 2022). MA NPI information came primarily from clinician NPIs on the base files (76.0% in 2022), with some organization NPIs used (23.8%). Recall that some of these organization NPIs are actually individual clinician NPIs. The bottom panel of Figure 1 details the source of specialty code information. Almost all TM claims had specialty code already present on the base or line file (98.8% in 2022), with a small fraction supplemented through a match of NPI to the MD‐PPAS file (0.8%). In MA, most specialty codes were determined by matching to the MD‐PPAS file (87.7% in 2022). The remaining specialty codes were obtained through the taxonomy code crosswalk (12.2%).

**FIGURE 2:**
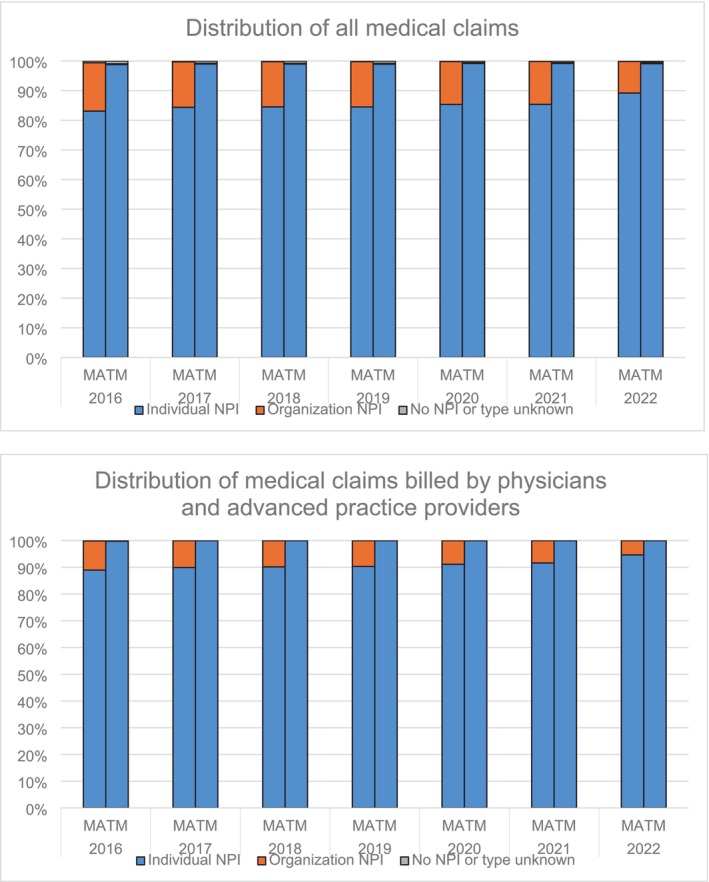
Distribution of identified NPI type. MA, Medicare Advantage; NPI, National Provider Identifier; TM, Traditional Medicare.

We used organization NPI when no source of individual clinician NPI was available. This increases the completeness of NPIs and specialty codes, but may not be useful in some contexts, such as when external data sources containing only individual clinicians need to be used. However, more than half of the NPIs pulled from organization‐level variables are actually individual NPIs. We merged our list of medical claims with the NPPES data, using the NPPES provider type code to determine the actual type of provider—individual or organization. The results are summarized in the top panel of Figure 2, showing that 83% of MA medical claims in 2016 had individual NPIs, improving to 89% in 2022. As we reviewed the specialty codes for organization NPIs, we found that many of these claims were billed by chiropractic and physical therapy practices. When we restrict medical claims to those billed by physicians and advanced practice providers, as shown in the bottom panel of Figure 2, the fraction with individual NPIs increases to 89% in 2016 and 95% in 2022.

#### 3.1.2 Attribution to Usual Source of Care

**FIGURE 3:**
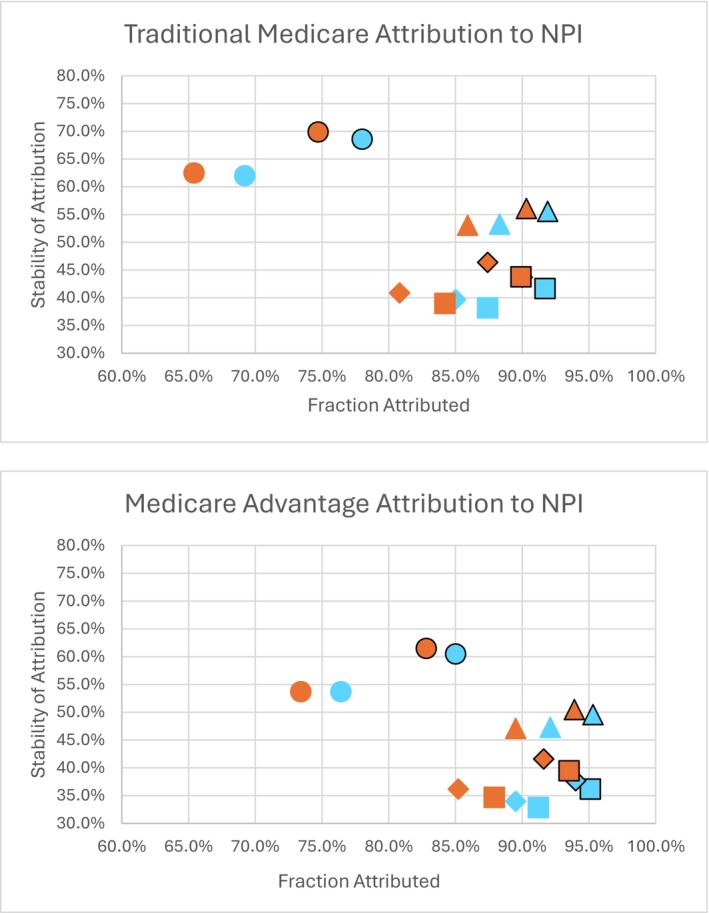
Patient attribution performance by plan type. NPI, National provider identifier. Symbol shape indicates provider types used in patient attribution to usual source of care: Physicians (MDs) and advanced practice providers (APPs), squares; MDs only, diamonds; primary care providers (PCPs) only, circles; PCPs then all MDs/APPs, triangles. Symbol color indicates encounter types used in patient attribution to usual source of care: Blue, all medical encounters; orange, evaluation and management encounters only. Outline indicates years of data used: Matching outline, current year only; black outline, lookback to prior year if current year does not result in attribution.

Figure 3 shows the findings on patient attribution, displaying the fraction attributed and attribution stability for each of the 16 algorithms for 2022 encounters. In this figure, clinician type is indicated by symbol shape, encounter type by symbol color, and the use of lookback by symbol outline. The highest fraction attributed occurs when we use all medical encounters with all MDs and APPs (blue squares), maximizing the chance of observing any patient‐clinician encounter. The lowest fraction attributed occurs when data are restricted to E&M visits with PCPs (orange circles). We hypothesize that the more restrictive data are less subject to spurious attribution caused by short‐term acute and specialty care needs. Consistent with this hypothesis, attribution stability is highest for the most restrictive algorithms (orange circles), while the broadest algorithms (blue squares) show lower attribution stability, illustrating the inherent tension between fraction attributed and stability. Differences between algorithms that use MDs and APPs (squares) versus MDs only (diamonds) are slight. Our fourth option for scope of encounters—using PCP encounters, if possible, then all MD/APP encounters if there is no PCP‐based attribution (triangles)—by definition meets or exceeds the fraction attributed by all MDs/APPs methods, while benefiting from some of the stability of PCP‐only attribution.

The anticipated increase in stability due to restricting data to E&M encounters (orange symbols) is slight and often is outweighed by smaller fractions attributed, relative to analogous methods using all medical encounters (blue symbols). When 2 years of data are available, methods incorporating a prior‐year lookback (black outlines) have a 5–10 percentage point increase in the fraction attributed relative to single‐year methods (matching outlines). Any increase in stability due to the lookback is largely tautological, as bringing the prior year's attribution forward will necessarily create consistency from year to year.

Overall, the fraction attributed is 3–8 percentage points higher in MA than in TM, especially for PCP‐only methods. Consistent with this increased fraction, stability in MA is 4–8 percentage points lower. We find very similar patterns in the MA and TM data when looking at patterns by algorithm. The only notable exception is a narrowing gap between methods using all medical encounters (blue symbols) versus E&M encounters (orange symbols) in MA.

As noted above, the PCP‐then‐all method (triangles) provides the fraction attributed found in methods using encounters with all physicians and APPs (squares), while benefiting from some of the stability of PCP‐only methods (circles). In addition, differences in fraction attributed between MA and TM are minimized when comparing the PCP‐then‐all algorithm using all medical claims with a lookback (blue triangles with black outline). The fraction attributed is similar (91.9% TM, 95.3% MA) and the differences in attribution stability are moderated (55.6% TM, 49.6% MA).

**FIGURE 4:**
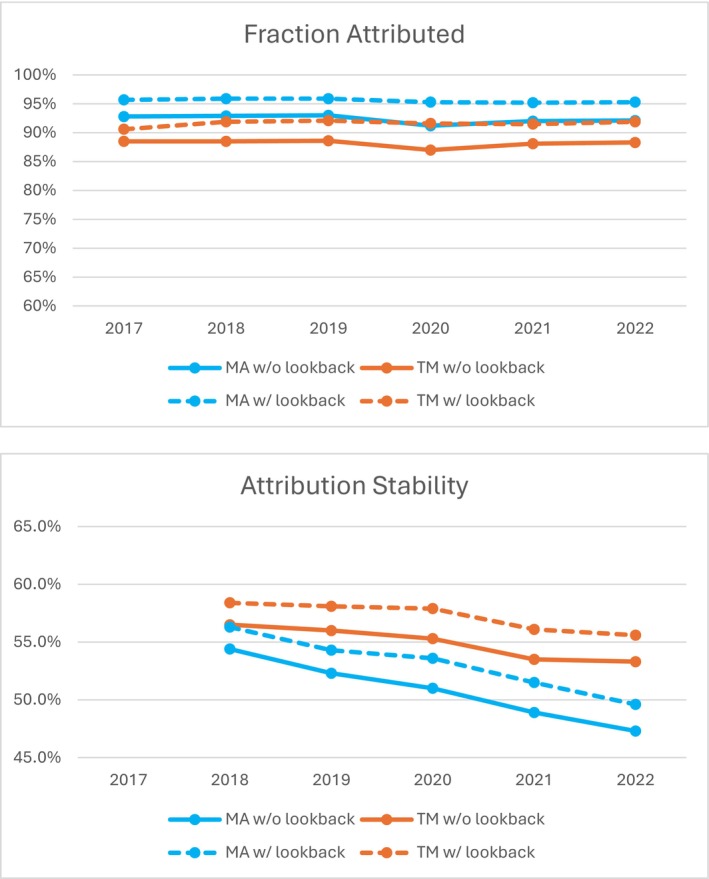
Fraction attributed and stability over time. MA, Medicare Advantage; TM, Traditional Medicare. Patient attribution to usual source of care is based on all medical encounters from primary care physicians, then from all medical encounters from physicians and advanced practice providers if initial attribution is unsuccessful.

Figure 4 shows trends in fraction attributed and stability for the PCP‐then‐all algorithms using medical claims. The top panel displays fraction attributed for 2017 through 2022, with 2016 used for the lookback algorithms. The bottom panel shows attribution stability for 2018 through 2022, compared to 2017 through 2021. The fraction attributed is very stable over time, remaining in the 87%–93% range. Relativities between MA and TM breadth are stable, with and without the lookback. However, stability declines for TM beneficiaries (from 57%–58% in 2018 to 53%–57% in 2022) and more steeply for MA enrollees (from 54%–56% in 2018 to 47%–50% in 2022). Patterns of stable fraction attributed and declining stability are similar when the algorithms use only E&M encounters (not shown).

Because our provider data enhancement uses organization NPIs, some MA beneficiaries will be attributed to an organization NPI rather than a clinician NPI. Across all attribution methods, 8%–10% of MA beneficiaries were attributed to organization NPIs during 2017–2021, decreasing to 4%–6% in 2022 as MA NPI data quality increased.

### 3.2 Robustness Testing

Detailed results of our robustness checks are in the Supporting Information. We note a few highlights here.

Eliminating the resource intensity tiebreaker from our baseline methods can reduce the fraction attributed by as little as 4–7 percentage points (PCP‐based methods) or as much as 17–20 percentage points (all MDs/APPs) (Exhibit S21) before the impact of the lookback. Attribution using resource intensity first, with the number of encounters as a tiebreaker, yields fraction attributed and stability nearly identical to our baseline count‐based methods (Exhibit S22).

Splitting MA contracts by data completeness shows little loss in fraction attributed, except for the PCP‐only E&M encounters method (Exhibit S24, orange circles). Stability declines by 1–5 percentage points for incomplete MA contracts, suggesting minor spurious attribution from missing data.

Fraction attributed and stability both increase from the first through third health risk quartile, with very little difference between the third and fourth quartiles (Exhibit S26). This suggests that a threshold effect may exist: nearly every beneficiary has a source of care once his or her health risk reaches a certain level. Slopes are slightly higher in TM than in MA populations.

MA fraction attributed is insensitive to the requirement that a minimum of 50% of an individual's encounters be mapped to provider NPI and specialty (Exhibit S27). The minor differences between MA and TM fraction attributed are driven almost entirely by differences in the rate of observing *any* patient‐provider encounters rather than this completeness requirement.

Finally, attribution of cancer patients to oncologists found consistent patterns in fraction attributed and the impact of a prior‐year lookback between MA and TM. Attribution rates were highly sensitive to the type of cancer (from 59%–70% for lung to 25%–34% for prostate), due to variations in intensity of treatment and the role of primary care providers in monitoring cancer status.

## 4 Discussion

We have demonstrated that parity can be achieved in clinician data quality between MA encounter and TM claims data. With our proposed methods, over 99% of MA medical claims have an assigned NPI and CMS specialty code. While some of the NPIs assigned to MA claims are organization rather than individual NPIs, by 2022 89% are individual NPIs, or 95% when examining claims billed by physicians or APPs.

Using this enriched clinician information, most methods for attributing patients to their usual source of care have similar performance in both coverage populations. We find slightly higher rates of attribution (3–8 percentage points) in the MA population, primarily because MA enrollees more frequently have at least one encounter with physicians or APPs. This is consistent with the managed care strategies of regular patient contact for preventive care and chronic condition management, ideally present in MA plans. Similarly, attribution stability is 4–8 percentage points lower among MA enrollees relative to TM beneficiaries. While this finding might indicate higher spurious attribution among MA enrollees, the fact that relative stability declines even in the restrictive PCP‐only algorithms suggests that it reflects more frequent changes in clinician affiliation rather than spurious attribution. This is consistent with an expectation that patients who are willing to accept limited provider networks may have weaker clinician ties than those who prefer non‐network plans such as TM.

Regardless of coverage type, for most purposes requiring patient attribution to a clinician, we recommend algorithms that identify the usual clinician based on PCP encounters, and then all physician and APP encounters if attribution is not possible using PCP encounters. These methods match the fraction attributed when all physician and APP encounters are used, with much of the stability of PCP‐only methods. Focusing on these methods, we examine trends in the fraction attributed and stability over time. The fraction attributed is consistent over time, even in MA where the early years of encounter data had more missing clinician and encounter information. However, stability is declining over time. Because this decline is consistent across coverage type, regardless of whether all medical encounters or more restrictive E&M encounters are used, it suggests that patient‐clinician affiliation is truly weakening over time. This has implications for providers' ability to recoup their investment in preventive care efforts through reduced patient needs in future years.

While we focus on the PCP‐then‐all algorithms, the context of use may require a different approach, such as attribution to a particular specialty to study the management of a condition such as cancer [34], or attribution at the HCO level, as in the Medicare Shared Savings Program [18]. Our robustness checks, including an example of attribution to oncologic specialists, provide some guidance for how methodological changes may affect the performance of the attribution algorithm, but this is an area for further study.

Our proposed methods of imputing clinician data can be adapted to other settings where NPI and/or taxonomy code data are available without specialty codes. Users can leverage publicly available NPPES and specialty crosswalk files to determine CMS specialty codes when an NPI‐to‐specialty mapping, such as CMS's MD‐PPAS file, is not available. Sample SAS code available on GitHub [https://github.com/CarolineCarlin/Attribution] provides a pathway for adapting both NPI/specialty determination and attribution methodologies to a wide variety of uses.

Our analysis has some limitations. Our measures of fraction attributed and attribution stability are merely suggestive of algorithm performance. Without a gold standard for identifying the patient's usual source of care, we cannot compute the true sensitivity of the methods for detecting the usual source of care, and the true measures of spurious attribution. In addition, we focus on data available for Medicare beneficiaries, with an emphasis on comparing the performance of common clinician attribution approaches across MA and TM populations. Results in other settings, particularly in younger age groups, may be different.

## Conflicts of Interest

The authors declare no conflicts of interest.

## Supporting information


**Data S1:** Supporting Information.

## Data Availability

Data sharing not applicable to this article as no datasets were generated during the current study. SAS code and reference files will be published on GitHub to allow users to apply proposed methods.
