# Supplementary material for: Provider Attribution in Medicare: Challenges and Solutions
Source: Health Serv Res. 2025 Oct 28;61(1):e70062. doi: 10.1111/1475-6773.70062 (PMC12857508; doi:10.1111/1475-6773.70062)
Supplement: Supplementary file 1 — Data S1: Supporting Information. [file HESR-61-e70062-s001.docx]

**Supplement to**

**“Provider Attribution in Medicare:**

**Challenges and Solutions”**

Caroline S. Carlin, PhD^1^

ccarlin@umn.edu

Roger Feldman, PhD^2^

feldm002@umn.edu

Jeah Jung, PhD^3^

jjung41@gmu.edu

October 16, 2025

^1^ Department of Family Medicine and Community Health, University of Minnesota, Minneapolis, MN

^2^ Division of Health Policy and Management, University of Minnesota, Minneapolis, MN

^3^ Department of Health Administration and Policy, George Mason University, Fairfax, VA

Corresponding author:

Caroline S. Carlin, PhD

Department of Family Medicine and Community Health

MMC 381

516 Delaware St. SE

Minneapolis, MN 55455

ccarlin@umn.edu

Acknowledgements:

This study was supported by the National Institute on Aging (1R01AG069352). The content is solely the responsibility of the authors and does not necessarily represent the views of the funder.

Contents

[Identification of Clinician Characteristics 5](#_Toc210226928)

[Exhibit S1 – Mapping of CMS Specialty Codes 7](#_Toc210226929)

[Completeness of Medicare Encounter Data Submission 11](#_Toc210226930)

[Study Sample Identification 12](#_Toc210226931)

[Detailed Demographic Summaries 13](#_Toc210226932)

[Exhibit S2 – Patient Characteristics of the Full Sample 14](#_Toc210226933)

[Exhibit S3 – Patient Characteristics for Attributed Population 1 15](#_Toc210226934)

[Exhibit S4 – Patient Characteristics for Attributed Population 2 16](#_Toc210226935)

[Exhibit S5 – Patient Characteristics for Attributed Population 3 17](#_Toc210226936)

[Exhibit S6 – Patient Characteristics for Attributed Population 4 18](#_Toc210226937)

[Exhibit S7 – Patient Characteristics for Attributed Population 5 19](#_Toc210226938)

[Exhibit S8 – Patient Characteristics for Attributed Population 6 20](#_Toc210226939)

[Exhibit S9 – Patient Characteristics for Attributed Population 7 21](#_Toc210226940)

[Exhibit S10 – Patient Characteristics for Attributed Population 8 22](#_Toc210226941)

[Exhibit S11 – Patient Characteristics for Attributed Population 9 23](#_Toc210226942)

[Exhibit S12 – Patient Characteristics for Attributed Population 10 24](#_Toc210226943)

[Exhibit S13 – Patient Characteristics for Attributed Population 11 25](#_Toc210226944)

[Exhibit S14 – Patient Characteristics for Attributed Population 12 26](#_Toc210226945)

[Exhibit S15 – Patient Characteristics for Attributed Population 13 27](#_Toc210226946)

[Exhibit S16 – Patient Characteristics for Attributed Population 14 28](#_Toc210226947)

[Exhibit S17 – Patient Characteristics for Attributed Population 15 29](#_Toc210226948)

[Exhibit S18 – Patient Characteristics for Attributed Population 16 30](#_Toc210226949)

[Changing Attribution Level 31](#_Toc210226950)

[Exhibit S19 – Attribution to Tax Identification Number 32](#_Toc210226951)

[Exhibit S20 – Attribution to Tax Identification Number and then Provider 34](#_Toc210226952)

[Attribution without Resource Intensity Tiebreaker 36](#_Toc210226953)

[Exhibit S21 – Elimination of Resource Intensity Tiebreaker 37](#_Toc210226954)

[Exhibit S22 – Change in Fraction Attributed Due to Elimination of Resource Use Tiebreaker 39](#_Toc210226955)

[Attribution Using Resource Intensity Rather than Count of Encounters 40](#_Toc210226956)

[Exhibit S23 – Attribution Using Resource Intensity Rather than Count of Encounters 41](#_Toc210226957)

[Impact of Complete Medicare Advantage Data Submission 43](#_Toc210226958)

[Exhibit S24 – Robustness of Attribution to Incomplete Medicare Encounter Data 44](#_Toc210226959)

[Treatment of Advanced Practice Providers as Primary Care Providers 46](#_Toc210226960)

[Exhibit S25 – Impact of Including Advanced Practice Providers with Primary Care Physicians 47](#_Toc210226961)

[Attribution Across Health Quartiles 49](#_Toc210226962)

[Exhibit S26 – Attribution by Health Risk Quartile 50](#_Toc210226963)

[Impact of Minimum Provider Information Requirement 51](#_Toc210226964)

[Exhibit S27 – Reasons for Lack of Attribution by Minimum Data Requirement 53](#_Toc210226965)

[Exhibit S28 – Increasing Minimum Provider Data Requirement from 50% to 100% of Encounters 54](#_Toc210226966)

[Attribution to Specialty Clinicians 56](#_Toc210226967)

[Exhibit S29 – Oncology Attribution 58](#_Toc210226968)

[Distribution of Clinicians by Specialty After Data Enhancement 59](#_Toc210226969)

[Exhibit S30 – Distribution of Medical Claims by Type of Specialty 60](#_Toc210226970)

[Guidelines for Sample SAS Code 61](#_Toc210226971)

[Data Preparation 61](#_Toc210226972)

[Overview of Attribution Code 62](#_Toc210226973)

[Autoexec Programs 63](#_Toc210226974)

[Cohort Selection and Data Preparation 63](#_Toc210226975)

[Visit-based Attribution with Tiebreaker 64](#_Toc210226976)

[Cost-based Attribution with Tiebreaker 65](#_Toc210226977)

[Visit-based Attribution with No Tiebreaker 66](#_Toc210226978)

[References 67](#_Toc210226979)

# Identification of Clinician Characteristics

We extracted Outpatient Facility claims for medical encounters, and Carrier claims for medical encounters in outpatient places of service. Medical encounters were defined as claims with Healthcare Common Procedure Coding System (HCPCS) codes in the range 90281 – 99607. Outpatient places of service included claims with place of service codes in the list 05, 06, 07, 08, 11, 12, 13, 14, 33, 19, 22, 49, 50, 71, or 72. For these encounters, we assigned clinician Centers for Medicare and Medicaid Services (CMS) specialty codes using the following hierarchical algorithm in both Medicare Advantage (MA) and Traditional Medicare (TM) data, though TM claims rarely made it past the first two steps in the hierarchy.

- Use performing provider National Provider Identifier (NPI) from Carrier line file, mapped to Medicare Data on Provider Practice and Specialty (MD-PPAS) to obtain CMS specialty code when not already present on the file.
- Use attending provider NPI from the Outpatient base file or rendering provider NPI from Carrier base file, mapped to MD-PPAS to obtain CMS specialty code when not already present on the file.
- Use organizational NPI mapped to MD-PPAS to obtain CMS specialty code.
- Use organizational NPI’s taxonomy code mapped to CMS specialty code using the taxonomy-specialty crosswalk.
- If organizational NPI is present but no taxonomy code is provided, map NPI to taxonomy using year-end National Plan & Provider Enumeration System (NPPES) files,^1^ and then to CMS specialty code using the taxonomy-specialty crosswalk.

When a Carrier claim is in a place of service eligible for an Outpatient Facility claim,^2^ and there is a match of beneficiary, procedure code and date service with an Outpatient Facility claim, we give preference to the specialty information present on the Carrier file for that encounter. We mapped the CMS specialty codes to following designations: primary care physician (PCP), specialty care physician (SCP), advanced practice provider (APP, including nurse practitioners and physician assistants), or other clinicians (e.g., chiropractors, physical therapists), as shown in Exhibit S1.

The taxonomy-specialty crosswalk was developed using a combination of CMS crosswalks published in 2017^3^ and 2025,^4^ to capture emerging specialties over time. When a taxonomy was mapped to multiple specialties, the most prevalent specialty for that taxonomy in our TM study sample was used. Some taxonomies in the NPPES coding system were not included in the crosswalks. If their specialties could not be imputed by our TM study sample, they were mapped to dummy CMS specialty codes ZF (unmapped facility) and ZP (unmapped professional), and their encounters classified as having no clinician information. The complete mapping is available with the sample attribution SAS-based software available at <https://github.com/CarolineCarlin/Attribution.git>.

## Exhibit S1 – Mapping of CMS Specialty Codes

| ***Code*** | ***Description*** | ***Provider Type*** |
| --- | --- | --- |
| 00 | Carrier wide | Other |
| 01 | General practice | PCP |
| 02 | General surgery | SCP |
| 03 | Allergy/immunology | SCP |
| 04 | Otolaryngology | SCP |
| 05 | Anesthesiology | SCP |
| 06 | Cardiology | SCP |
| 07 | Dermatology | SCP |
| 08 | Family practice | PCP |
| 09 | Interventional pain management | SCP |
| 10 | Gastroenterology | SCP |
| 11 | Internal medicine | PCP |
| 12 | Osteopathic manipulative therapy | SCP |
| 13 | Neurology | SCP |
| 14 | Neurosurgery | SCP |
| 15 | Speech language pathologists | SCP |
| 16 | Obstetrics/gynecology | SCP |
| 17 | Hospice and palliative care | SCP |
| 18 | Ophthalmology | SCP |
| 19 | Oral surgery (dentists only) | Other |
| 20 | Orthopedic surgery | SCP |
| 21 | Cardiac electrophysiology | SCP |
| 22 | Pathology | SCP |
| 23 | Sports medicine | SCP |
| 24 | Plastic and reconstructive surgery | SCP |
| 25 | Physical medicine and rehabilitation | SCP |
| 26 | Psychiatry | SCP |
| 27 | Geriatric psychiatry | SCP |
| 28 | Colorectal surgery (formerly proctology) | SCP |
| 29 | Pulmonary disease | SCP |
| 30 | Diagnostic radiology | SCP |
| 31 | Intensive cardiac rehabilitation | SCP |
| 32 | Anesthesiologist assistants (eff. 4/1/03) | Other |
| 33 | Thoracic surgery | SCP |
| 34 | Urology | SCP |
| 35 | Chiropractic | Other |
| 36 | Nuclear medicine | SCP |
| 37 | Pediatric medicine | PCP |
| 38 | Geriatric medicine | PCP |
| 39 | Nephrology | SCP |
| 40 | Hand surgery | SCP |
| 41 | Optometrist | SCP |
| 42 | Certified nurse midwife | Other |
| 43 | CRNA | Other |
| 44 | Infectious disease | SCP |
| 45 | Mammography screening center | Other |
| 46 | Endocrinology | SCP |
| 47 | Independent diagnostic testing facility | Other |
| 48 | Podiatry | Other |
| 49 | Ambulatory surgical center | SCP |
| 50 | Nurse practitioner | APP |
| 51 | Medical supply company with certified orthotist | Other |
| 52 | Medical supply company with certified prosthetist | Other |
| 53 | Medical supply company with certified prosthetist-orthotist | Other |
| 54 | Medical supply company not included in 51, 52, or 53 | Other |
| 55 | Individual certified orthotist | Other |
| 56 | Individual certified prosthetist | Other |
| 57 | Individual certified prosthetist-orthotist | Other |
| 58 | Medical supply company with registered pharmacist | Other |
| 59 | Ambulance service supplier | Other |
| 60 | Public health or welfare agencies | Other |
| 61 | Voluntary health or charitable agencies | Other |
| 62 | Psychologist | Other |
| 63 | Portable X-ray supplier | Other |
| 64 | Audiologist | Other |
| 65 | Physical therapist | Other |
| 66 | Rheumatology | SCP |
| 67 | Occupational therapist | Other |
| 68 | Clinical psychologist | Other |
| 69 | Clinical laboratory | Other |
| 70 | Multispecialty clinic or group practice | Other |
| 71 | Registered dietician/nutrition professional | Other |
| 72 | Pain management | Other |
| 73 | Mass immunization roster biller | Other |
| 74 | Radiation therapy centers | Other |
| 75 | Slide preparation facilities | Other |
| 76 | Peripheral vascular disease | SCP |
| 77 | Vascular surgery | SCP |
| 78 | Cardiac surgery | SCP |
| 79 | Addiction medicine | SCP |
| 80 | Licensed clinical social worker | Other |
| 81 | Critical care (intensivists) | SCP |
| 82 | Hematology | SCP |
| 83 | Hematology/oncology | SCP |
| 84 | Preventive medicine | PCP |
| 85 | Maxillofacial surgery | SCP |
| 86 | Neuropsychiatry | SCP |
| 87 | All other suppliers (e.g. drug and department stores) | Other |
| 88 | Unknown supplier/provider specialty | Other |
| 89 | Certified clinical nurse specialist | Other |
| 90 | Medical oncology | SCP |
| 91 | Surgical oncology | SCP |
| 92 | Radiation oncology | SCP |
| 93 | Emergency medicine | SCP |
| 94 | Interventional radiology | SCP |
| 95 | Medical supply company with certified prosthetist | Other |
| 96 | Optician | Other |
| 97 | Physician assistant | APP |
| 98 | Gynecologist/oncologist | SCP |
| 99 | Unknown physician specialty | SCP |
| A0 | Hospital (DMERCs only) | Other |
| A1 | SNF (DMERCs only) | Other |
| A2 | Intermediate care nursing facility (DMERCs only) | Other |
| A3 | Nursing facility, other (DMERCs only) | Other |
| A4 | HHA (DMERCs only) | Other |
| A5 | Pharmacy (DMERC) | Other |
| A6 | Medical supply company with respiratory therapist (DMERCs only) | Other |
| A7 | Department store (DMERC) | Other |
| A8 | Grocery store (DMERC) | Other |
| A9 | Indian Health Service (IHS), tribe and tribal organizations (non-hospital) | Other |
| B1 | Supplier of oxygen and/or oxygen related equipment | Other |
| B2 | Pedorthic personnel | Other |
| B3 | Medical supply company with pedorthic personnel | Other |
| B4 | Does not meet definition of health care provider | Other |
| B5 | Ocularist | Other |
| C0 | Sleep medicine | SCP |
| C1 | Centralized flu | Other |
| C2 | Indirect payment procedure | Other |
| C3 | Interventional cardiology | SCP |
| C4 | Restricted use | Other |
| C5 | Dentist | Other |
| C6 | Hospitalist | SCP |
| C7 | Advanced heart failure and transplant cardiology | SCP |
| C8 | Medical toxicology | SCP |
| C9 | Hematopoietic cell transplantation and cellular therapy | SCP |
| D1 | Medicare Diabetes Prevention Program | Other |
| D2 | In-person MDPP supplier with distance learning | Other |
| D3 | Medical genetics and genomics | SCP |
| D4 | Undersea and hyperbaric medicine | SCP |
| D5 | Opioid treatment program | Other |
| D6 | Home infusion therapy services | Other |
| D7 | Micrographic dermatologic surgery | SCP |
| D8 | Adult congenital heart disease | SCP |
| E1 | Marriage and family therapist | Other |
| E2 | Mental health counselor | Other |
| E3 | Dental anesthesiology | Other |
| E4 | Dental public health | Other |
| E5 | Endodontics | Other |
| E6 | Oral and maxillofacial pathology | Other |
| E7 | Oral and maxillofacial radiology | Other |
| ZF | _Unmapped facility or service (dummy code) | No descriptor |
| ZP | _Unmapped professional or support personnel (dummy code) | No descriptor |

PCP = primary care physician; SCP = specialty care physician; APP = advanced practice provider

# Completeness of Medicare Encounter Data Submission

Incomplete submission of patient encounters has been a concern since CMS originally released the 2015 data. While the quality of the data is improving over time, significant gaps still exist.^5^ Jung, Carlin, Feldman and Tran (2022)^6^ compared encounter data to two external sources: Medicare Provider Analysis and Review (MedPAR) files for inpatient rates, and Healthcare Effectiveness Data and Information System (HEDIS) data for outpatient care over the period 2015 to 2018. They found that 48% of contracts with at least 2500 enrollees submitted reasonably complete data in 2015, improving to 61% by 2018. While relevant HEDIS data are no longer released, we replicated their methods from 2015 to 2022 with MedPAR files only, finding 86% of contracts with at least 2500 enrollees had relatively complete inpatient data in 2015, improving to 90% in 2018 and 97% in 2022. If the completeness of outpatient encounters is improving at a similar pace, the probability of observing a complete picture of patient encounters is also increasing.

# Study Sample Identification

The primary data source was the CMS standard 20% sample of Medicare Advantage (MA) encounter and Traditional Medicare (TM) claims data,^7^ including 2016-2022 dates of service. We restricted the sample to beneficiaries with continuous MA or TM enrollment during the calendar year, full Part A and Part B coverage, and residence in the 50 states or the District of Columbia.

Full Part A and Part B coverage was established by looking at the Master Beneficiary Summary File (MBSF) variables capturing months of Part A and B coverage. In the year of entry, the months of coverage had to equal months from the coverage date through December 31, in the year of death months of coverage had to equal months from January 1 through date of death, otherwise the months of coverage had to equal 12.

Continuous MA coverage in the year was defined as having months of Part C coverage recorded on the MBSF equal to Part A coverage months. Continuous TM coverage was defined as having months of Part C coverage equal to zero. Those with mixed coverage were excluded.

Residence in the 50 states or the District of Columbia was determined using the state code available on the MBSF.

# Detailed Demographic Summaries

Summary statistics describing the MA and TM populations used in this study are available in the manuscript Exhibit 1, repeated here as Exhibit S2. We see that the MA population is more racially diverse, has a higher probability of participating in means-tested programs such as Medicaid and the low-income subsidy of copayments, has much higher rates of Part D coverage, and is less rural. Because the subset of patients that are attributed to provider may vary significantly from the overall population, we provide similar summaries for the attributed populations under each of the sixteen methods evaluated (Exhibits S3-S18).

We observe relatively small changes in demographics, even in the scenario where encounters are restricted to evaluation and management visits with a primary care physician (PCP) (Exhibit S5). In this scenario greater changes are observed in the TM population which has a mean age of 73.1 relative to the full sample mean age of 72.1. The MA mean age increase is smaller, to 73.1 from the full sample mean of 72.8. In Exhibit S5 we also see an increase in TM propensity to have Part D coverage (73.3% vs 69.0% in the full sample).

In the scenario with largest fraction attributed (Exhibit S14) -- where PCP medical encounters are used, then all medical encounters with physicians and advanced practice providers, supplemented with a lookback to prior year data -- differences are minimized. The mean age for the TM population is 72.6 relative to the full population mean of 72.1. For MA enrollees, this attributed population has a mean age of 72.9 relative to the full population mean of 72.8.

## Exhibit S2 – Patient Characteristics of the Full Sample


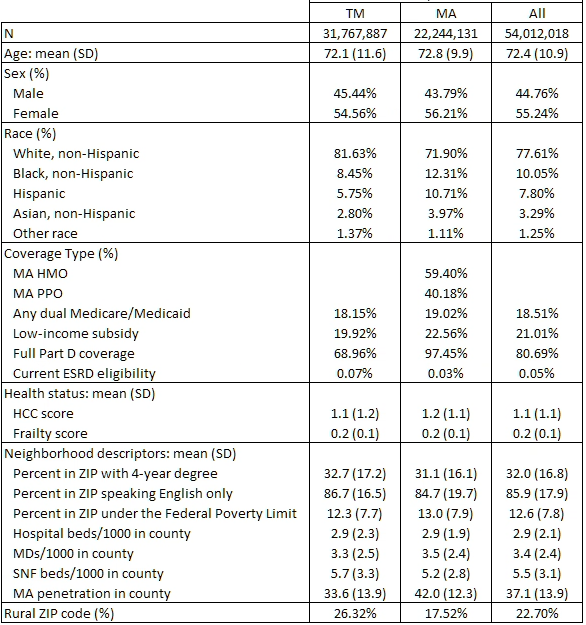


TM=Traditional Medicare; MA=Medicare Advantage; SD=standard deviation; HMO=health maintenance organization; PPO=preferred provider organization; ESRD=end-stage renal disease; HCC=hierarchical conditional category; MD=medical doctor; SNF=skilled nursing facility

## Exhibit S3 – Patient Characteristics for Attributed Population 1

Encounters include all medical encounters with physicians and advanced practice providers, with no lookback to prior year data (blue square with matching outline).


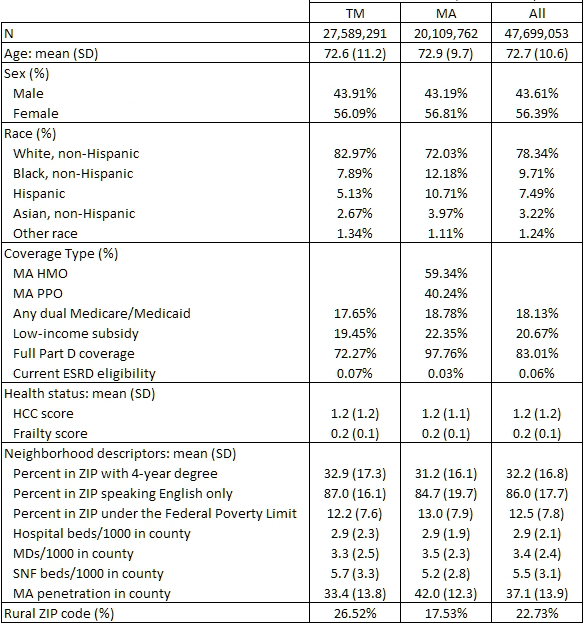


TM=Traditional Medicare; MA=Medicare Advantage; SD=standard deviation; HMO=health maintenance organization; PPO=preferred provider organization; ESRD=end-stage renal disease; HCC=hierarchical conditional category; MD=medical doctor; SNF=skilled nursing facility

## Exhibit S4 – Patient Characteristics for Attributed Population 2

Encounters include all medical encounters with physicians, with no lookback to prior year data (blue diamond with matching outline).


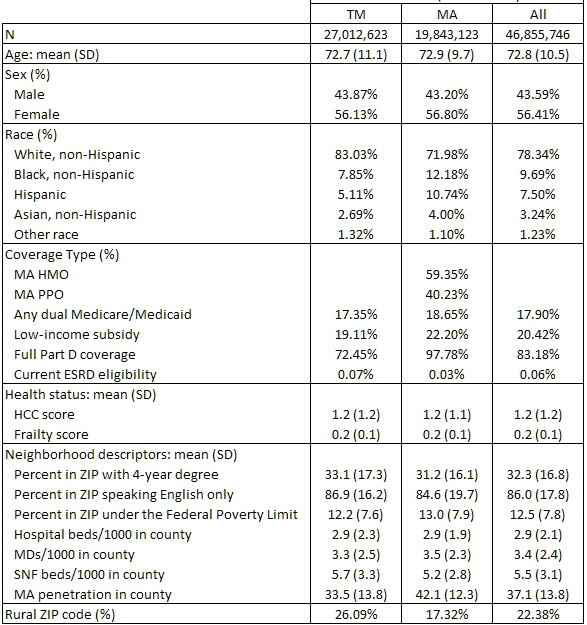


TM=Traditional Medicare; MA=Medicare Advantage; SD=standard deviation; HMO=health maintenance organization; PPO=preferred provider organization; ESRD=end-stage renal disease; HCC=hierarchical conditional category; MD=medical doctor; SNF=skilled nursing facility

## Exhibit S5 – Patient Characteristics for Attributed Population 3

Encounters include all medical encounters with primary care physicians, with no lookback to prior year data (blue circle with matching outline).


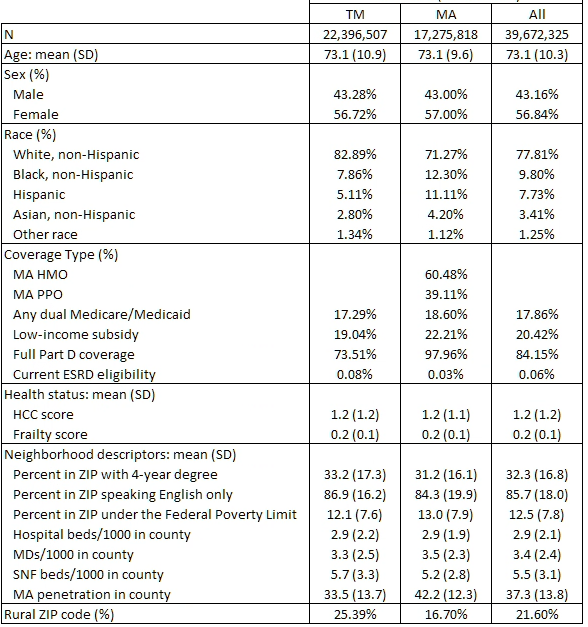


TM=Traditional Medicare; MA=Medicare Advantage; SD=standard deviation; HMO=health maintenance organization; PPO=preferred provider organization; ESRD=end-stage renal disease; HCC=hierarchical conditional category; MD=medical doctor; SNF=skilled nursing facility

## Exhibit S6 – Patient Characteristics for Attributed Population 4

Encounters include all medical encounters with primary care physicians, then all physicians and advanced practice providers, with no lookback to prior year data (blue triangle with matching outline).


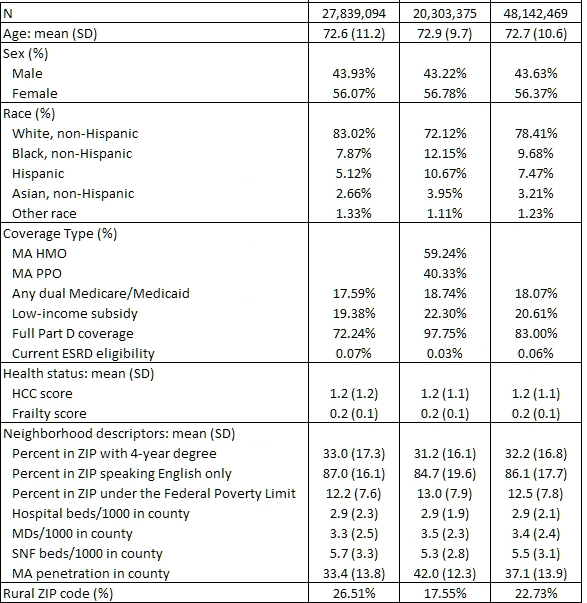


TM=Traditional Medicare; MA=Medicare Advantage; SD=standard deviation; HMO=health maintenance organization; PPO=preferred provider organization; ESRD=end-stage renal disease; HCC=hierarchical conditional category; MD=medical doctor; SNF=skilled nursing facility

## Exhibit S7 – Patient Characteristics for Attributed Population 5

Encounters include all evaluation and management encounters with physicians and advanced practice providers, with no lookback to prior year data (orange square with matching outline).


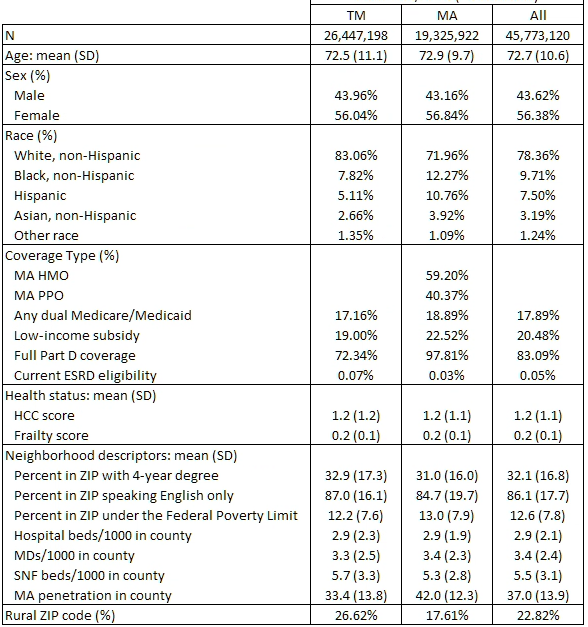


TM=Traditional Medicare; MA=Medicare Advantage; SD=standard deviation; HMO=health maintenance organization; PPO=preferred provider organization; ESRD=end-stage renal disease; HCC=hierarchical conditional category; MD=medical doctor; SNF=skilled nursing facility

## Exhibit S8 – Patient Characteristics for Attributed Population 6

Encounters include all evaluation and management encounters with physicians, with no lookback to prior year data (orange diamond with matching outline).


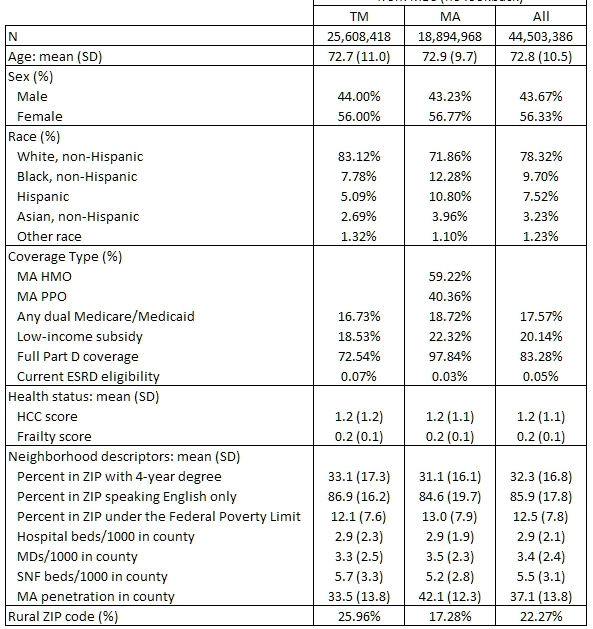


TM=Traditional Medicare; MA=Medicare Advantage; SD=standard deviation; HMO=health maintenance organization; PPO=preferred provider organization; ESRD=end-stage renal disease; HCC=hierarchical conditional category; MD=medical doctor; SNF=skilled nursing facility

## Exhibit S9 – Patient Characteristics for Attributed Population 7

Encounters include all evaluation and management encounters with primary care physicians, with no lookback to prior year data (orange circle with matching outline).


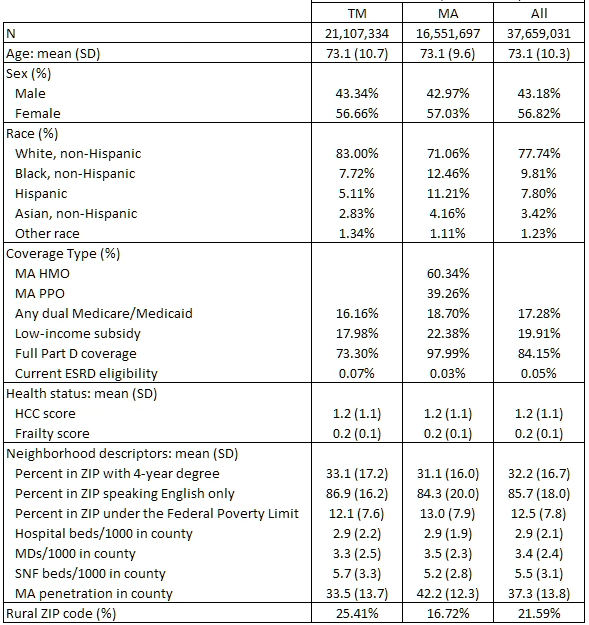


TM=Traditional Medicare; MA=Medicare Advantage; SD=standard deviation; HMO=health maintenance organization; PPO=preferred provider organization; ESRD=end-stage renal disease; HCC=hierarchical conditional category; MD=medical doctor; SNF=skilled nursing facility

## Exhibit S10 – Patient Characteristics for Attributed Population 8

Encounters include all evaluation and management encounters with primary care physicians, then all physicians and advanced practice providers, with no lookback to prior year data (orange triangle with matching outline).


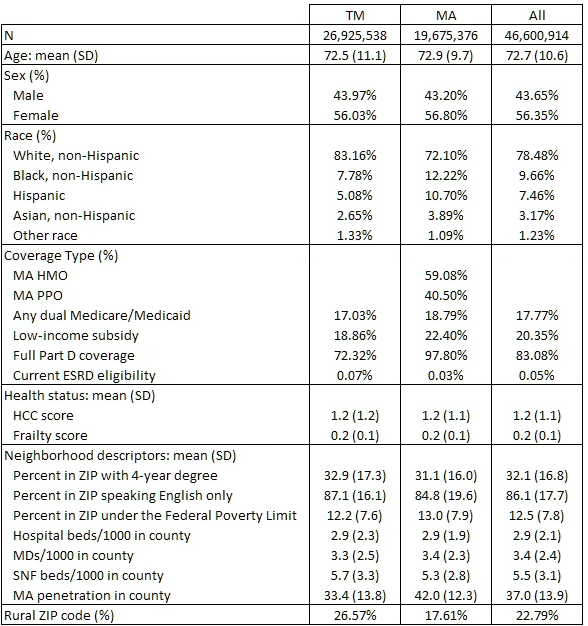


TM=Traditional Medicare; MA=Medicare Advantage; SD=standard deviation; HMO=health maintenance organization; PPO=preferred provider organization; ESRD=end-stage renal disease; HCC=hierarchical conditional category; MD=medical doctor; SNF=skilled nursing facility

## Exhibit S11 – Patient Characteristics for Attributed Population 9

Encounters include all medical encounters with physicians and advanced practice providers, with lookback to prior year data (blue square with black outline).


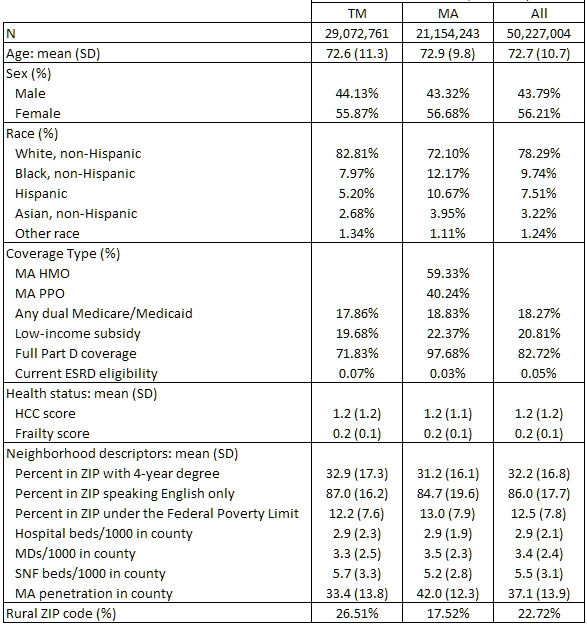


TM=Traditional Medicare; MA=Medicare Advantage; SD=standard deviation; HMO=health maintenance organization; PPO=preferred provider organization; ESRD=end-stage renal disease; HCC=hierarchical conditional category; MD=medical doctor; SNF=skilled nursing facility

## Exhibit S12 – Patient Characteristics for Attributed Population 10

Encounters include all medical encounters with physicians, with lookback to prior year data (blue diamond with black outline).


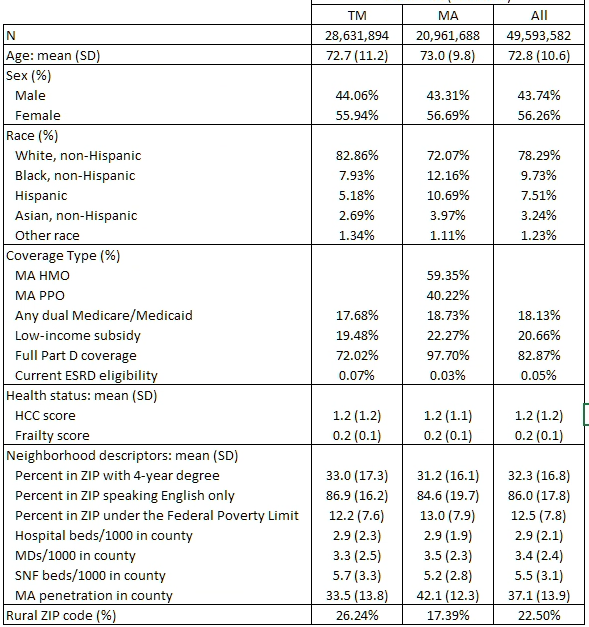


TM=Traditional Medicare; MA=Medicare Advantage; SD=standard deviation; HMO=health maintenance organization; PPO=preferred provider organization; ESRD=end-stage renal disease; HCC=hierarchical conditional category; MD=medical doctor; SNF=skilled nursing facility

## Exhibit S13 – Patient Characteristics for Attributed Population 11

Encounters include all medical encounters with primary care physicians, with lookback to prior year data (blue circle with black outline).


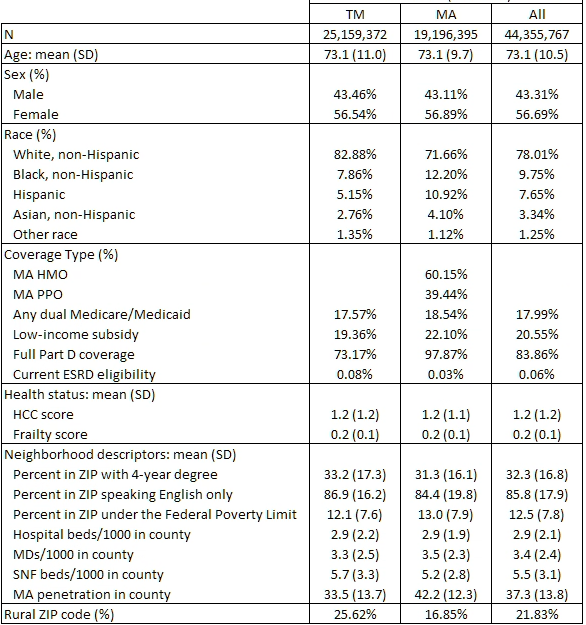


TM=Traditional Medicare; MA=Medicare Advantage; SD=standard deviation; HMO=health maintenance organization; PPO=preferred provider organization; ESRD=end-stage renal disease; HCC=hierarchical conditional category; MD=medical doctor; SNF=skilled nursing facility

## Exhibit S14 – Patient Characteristics for Attributed Population 12

Encounters include all medical encounters with primary care physicians, then all physicians and advanced practice providers, with lookback to prior year data (blue triangle with black outline).


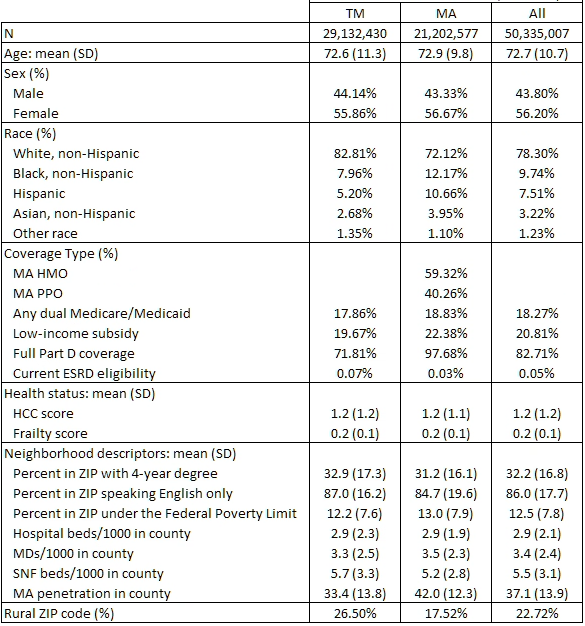


TM=Traditional Medicare; MA=Medicare Advantage; SD=standard deviation; HMO=health maintenance organization; PPO=preferred provider organization; ESRD=end-stage renal disease; HCC=hierarchical conditional category; MD=medical doctor; SNF=skilled nursing facility

## Exhibit S15 – Patient Characteristics for Attributed Population 13

Encounters include all evaluation and management encounters with physicians and advanced practice providers, with lookback to prior year data (orange square with black outline).


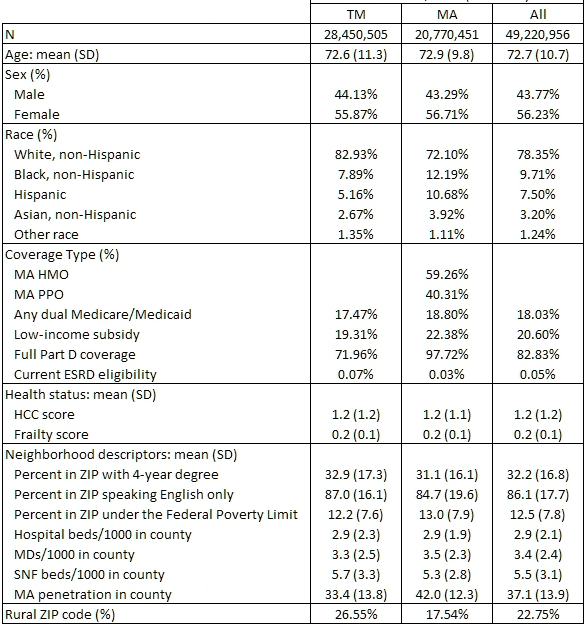


TM=Traditional Medicare; MA=Medicare Advantage; SD=standard deviation; HMO=health maintenance organization; PPO=preferred provider organization; ESRD=end-stage renal disease; HCC=hierarchical conditional category; MD=medical doctor; SNF=skilled nursing facility

## Exhibit S16 – Patient Characteristics for Attributed Population 14

Encounters include all evaluation and management encounters with physicians, with lookback to prior year data (orange diamond with black outline).


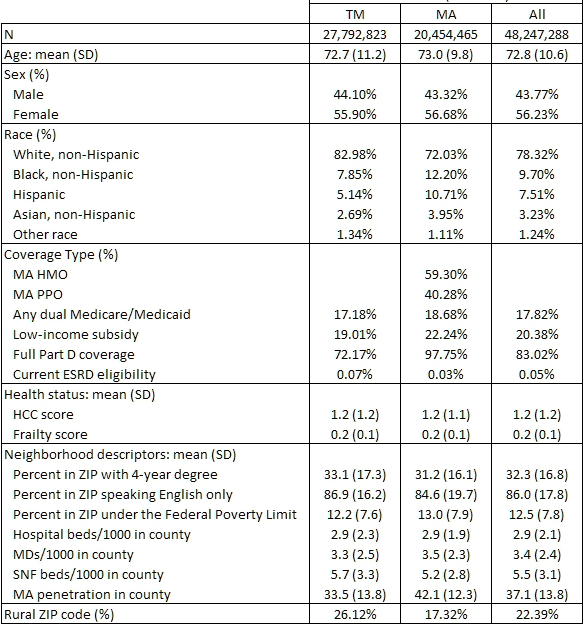


TM=Traditional Medicare; MA=Medicare Advantage; SD=standard deviation; HMO=health maintenance organization; PPO=preferred provider organization; ESRD=end-stage renal disease; HCC=hierarchical conditional category; MD=medical doctor; SNF=skilled nursing facility

## Exhibit S17 – Patient Characteristics for Attributed Population 15

Encounters include all evaluation and management encounters with primary care physicians, with lookback to prior year data (orange circle with black outline).


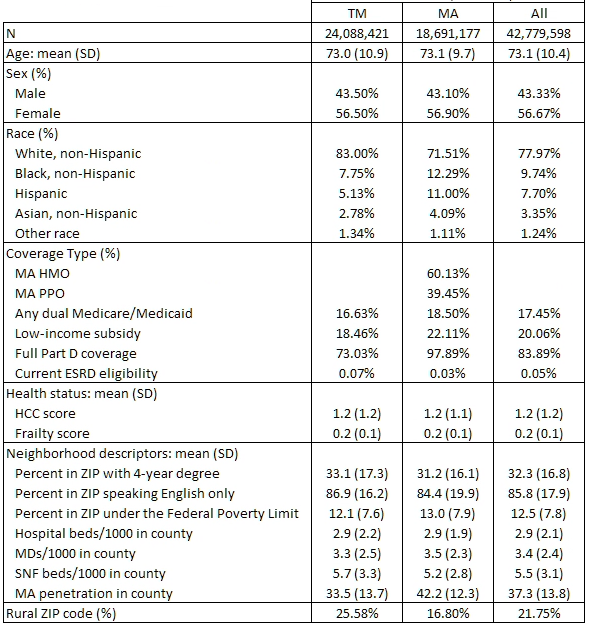


TM=Traditional Medicare; MA=Medicare Advantage; SD=standard deviation; HMO=health maintenance organization; PPO=preferred provider organization; ESRD=end-stage renal disease; HCC=hierarchical conditional category; MD=medical doctor; SNF=skilled nursing facility

## Exhibit S18 – Patient Characteristics for Attributed Population 16

Encounters include all evaluation and management encounters with primary care physicians, then all physicians and advanced practice providers, with lookback to prior year data (orange triangle with black outline).


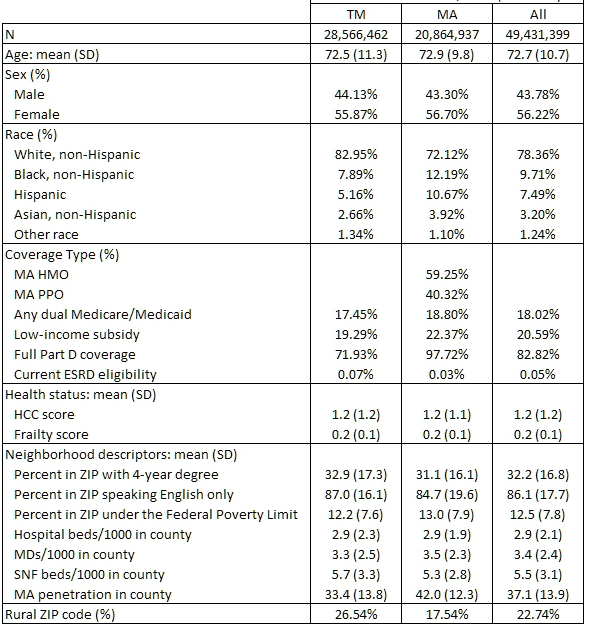


TM=Traditional Medicare; MA=Medicare Advantage; SD=standard deviation; HMO=health maintenance organization; PPO=preferred provider organization; ESRD=end-stage renal disease; HCC=hierarchical conditional category; MD=medical doctor; SNF=skilled nursing facility

# Changing Attribution Level

Our baseline methods focus on attribution directly to the national provider identifier (NPI) level. However, different financial, care management or research goals may make attribution at the health care organization (HCO) or practice location desirable. We can use tax identification number (TIN) in the claims data to approximate HCO level,^8^ although this should be done with caution, as large HCOs may use multiple TINs, or an acquired location may continue to bill under their legacy TIN long after the acquisition is concluded. In addition, using National Plan & Provider Enumeration System (NPPES) data it is possible to map NPIs to practice location for clinic site-level attribution, although significant address cleaning would be required.

For comparison, here we show our baseline NPI results next to results at the TIN level (Exhibit S19). Stability increases, as movement among NPIs within TIN does not disrupt the attribution, while the fraction attributed is strikingly similar to the NPI-level results.

We also tested whether stability could be improved by attributing to the TIN level, and then finding the plurality of visits at the NPI level within that TIN. There is no meaningful difference between these results and attributing directly to the NPI level (Exhibit S20).

## Exhibit S19 – Attribution to Tax Identification Number


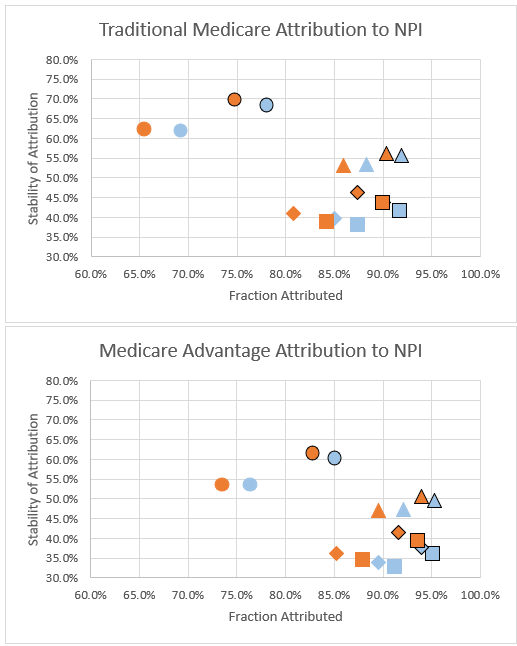

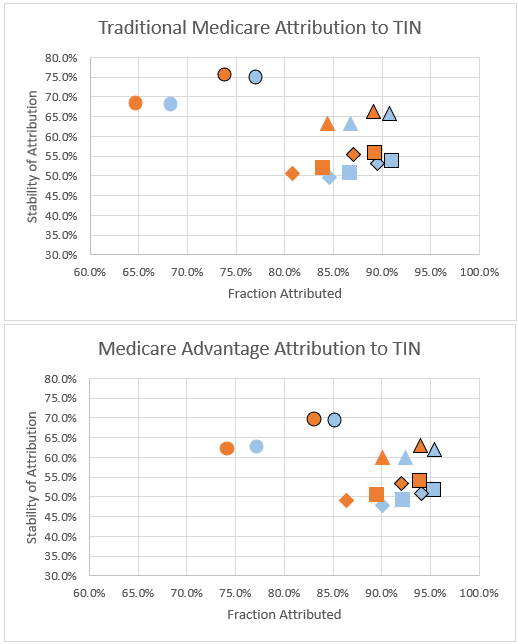


**Legend**

Symbol shape indicates provider types used in attribution: physicians (MDs) and advanced practice providers (APPs)=squares; MDs only=diamonds; primary care providers (PCPs) only=circles; PCPs then all MDs/APPs=triangles.

Symbol color indicates encounter types used in attribution: blue=all medical encounters; orange=evaluation and management encounters only.

Outline indicates years of data used: matching outline=current year only; black outline=lookback to prior year if current year doesn’t result in attribution.

TIN=tax identification number; NPI=national provider identifier

## Exhibit S20 – Attribution to Tax Identification Number and then Provider


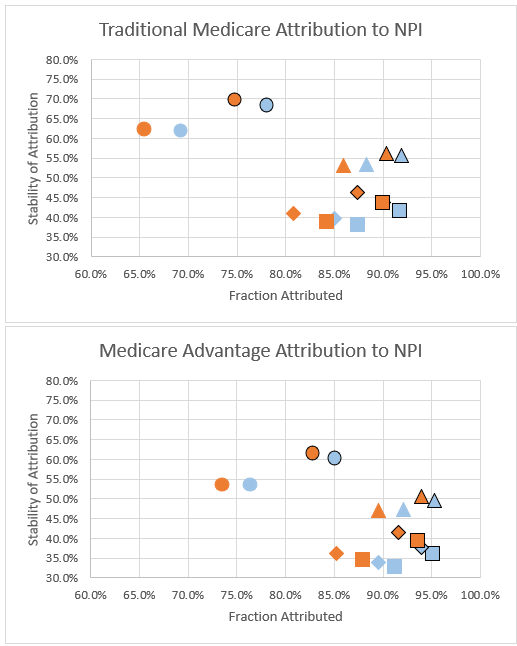

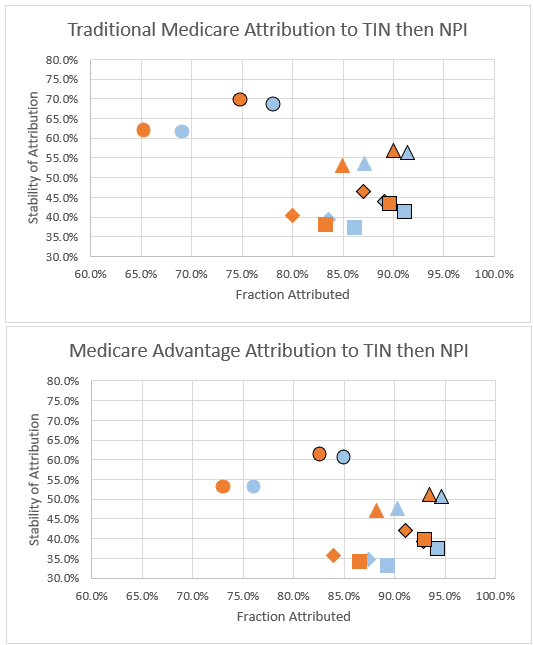


**Legend**

Symbol shape indicates provider types used in attribution: physicians (MDs) and advanced practice providers (APPs)=squares; MDs only=diamonds; primary care providers (PCPs) only=circles; PCPs then all MDs/APPs=triangles.

Symbol color indicates encounter types used in attribution: blue=all medical encounters; orange=evaluation and management encounters only.

Outline indicates years of data used: matching outline=current year only; black outline=lookback to prior year if current year doesn’t result in attribution.

TIN=tax identification number; NPI=national provider identifier

# Attribution without Resource Intensity Tiebreaker

Our methods use attribution to the provider with the plurality of encounters, with intensity of resources used in delivering care as a tiebreaker when two or more providers have equal pluralities of visits. Resource intensity is measured as the sum of standardized fees per Healthcare Common Procedure Coding System (HCPCS) code. But the computation of standardized fees is a complex process,^6^ and researchers may not have access to the necessary data. Here we document how the fraction attributed and attribution stability are impacted by eliminating this tiebreaker.

We find that methods focused on primary care providers (either PCP only [circles] or PCP then all physicians and advanced practice providers [triangles]) are more robust to this loss than methods that use all specialties, and that robustness is enhanced when lookback methods are used (Exhibit S21). In the worst-case scenario, using all specialties without a lookback (squares with matching outlines), the fraction attributed can drop by 19-20 percentage points when the tiebreaker is eliminated (shown in tabular form in Exhibit S22), compared to 4-7 percentage points for PCP-based methods without lookback. Methods without lookback (matching outlines) experience negligible change in stability of attribution. When a lookback method is used (black outlines), there is an increase in stability that is simply a reflection of bringing prior attribution forward more frequently, due to smaller fractions attributed in the current year.

## Exhibit S21 – Elimination of Resource Intensity Tiebreaker


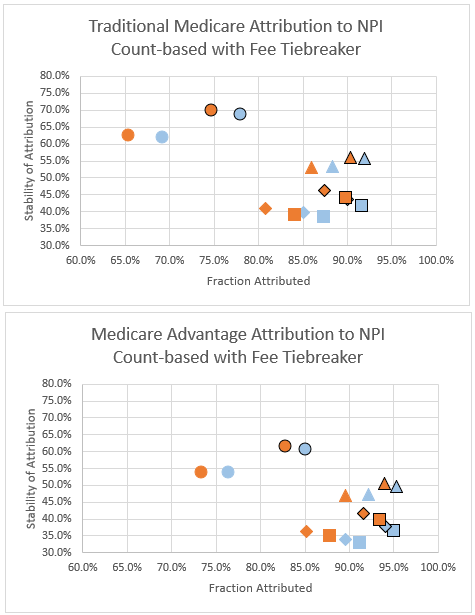

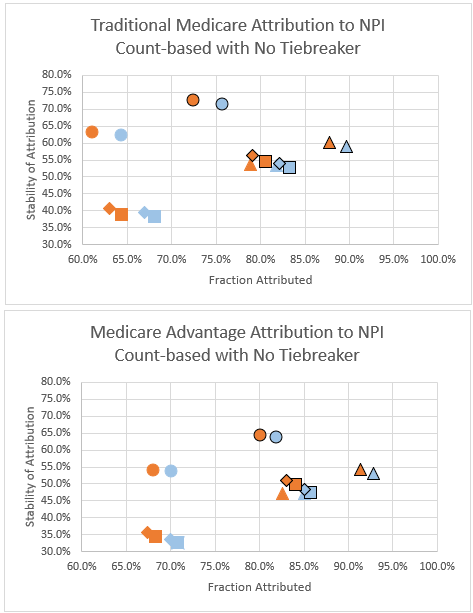


**Legend**

Symbol shape indicates provider types used in attribution: physicians (MDs) and advanced practice providers (APPs)=squares; MDs only=diamonds; primary care providers (PCPs) only=circles; PCPs then all MDs/APPs=triangles.

Symbol color indicates encounter types used in attribution: blue=all medical encounters; orange=evaluation and management encounters only.

Outline indicates years of data used: matching outline=current year only; black outline=lookback to prior year if current year doesn’t result in attribution.

NPI=National Provider Identifier

## Exhibit S22 – Change in Fraction Attributed Due to Elimination of Resource Use Tiebreaker


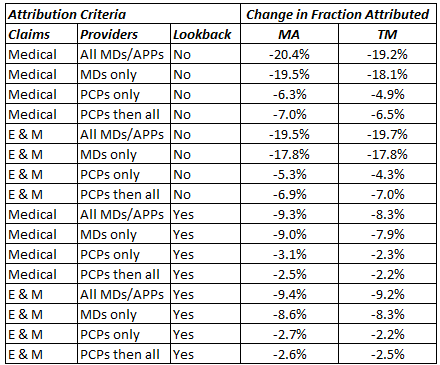


E&M = evaluation and management; MDs = physicians; PCPs = primary care physicians;

APPs = advanced practice providers; MA = Medicare Advantage; TM = Traditional Medicare

# Attribution Using Resource Intensity Rather than Count of Encounters

In some contexts, attribution by plurality of resource intensity (e.g., spending) rather than plurality of encounters may be desirable. Here we compare our baseline attribution using count of encounters (with a resource intensity tiebreaker) against attribution using resource intensity (with count of encounters as a tiebreaker). Resource intensity is measured as the sum of standardized fees per Healthcare Common Procedure Coding System (HCPCS) code.^6^ We find nearly identical fraction attributed and attribution stability as in our baseline calculations (Exhibit S23).

## Exhibit S23 – Attribution Using Resource Intensity Rather than Count of Encounters


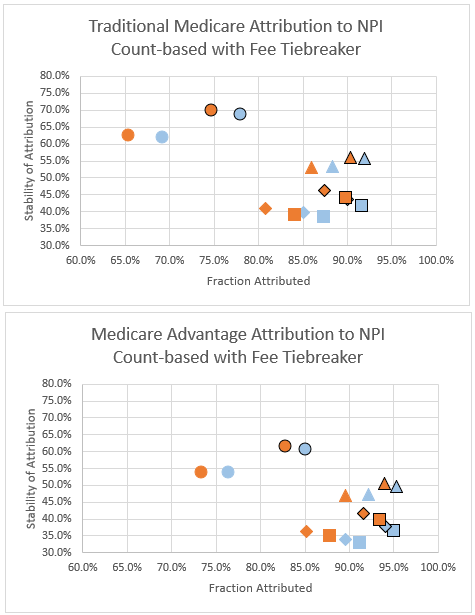

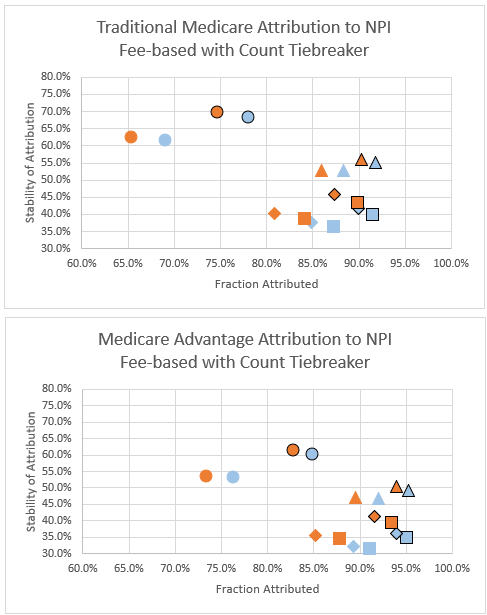


**Legend**

Symbol shape indicates provider types used in attribution: physicians (MDs) and advanced practice providers (APPs)=squares; MDs only=diamonds; primary care providers (PCPs) only=circles; PCPs then all MDs/APPs=triangles.

Symbol color indicates encounter types used in attribution: blue=all medical encounters; orange=evaluation and management encounters only.

Outline indicates years of data used: matching outline=current year only; black outline=lookback to prior year if current year doesn’t result in attribution.

NPI=National Provider Identifier

# Impact of Complete Medicare Advantage Data Submission

One concern about Medicare Advantage encounter data is the incomplete nature of the encounters submitted.^6,9^ If we are missing encounters, we would be more likely to be unable to complete the attribution, or possibly be more subject to spurious attribution reducing stability. Following previous work,^6^ we identify MA contracts with incomplete data submission and summarize the fraction attributed and attribution stability separately for enrollees in complete and incomplete contracts.

With incomplete data, we find some small fractions attributed, particularly for methods that rely only on primary care physician evaluation and management encounters (Exhibit S24). Similarly, the loss of stability is largely confined to methods that rely on primary care physician encounters (3-5 percentage-point decline).

##
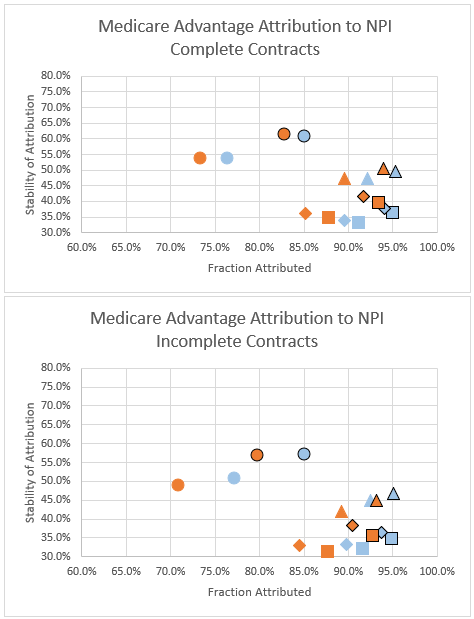
Exhibit S24 – Robustness of Attribution to Incomplete Medicare Encounter Data


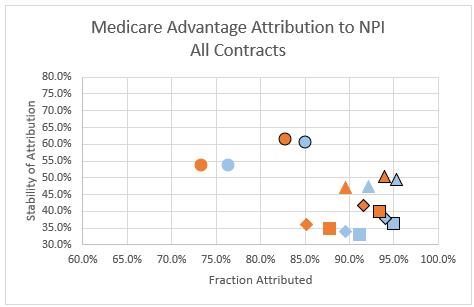


**Legend**

Symbol shape indicates provider types used in attribution: physicians (MDs) and advanced practice providers (APPs)=squares; MDs only=diamonds; primary care providers (PCPs) only=circles; PCPs then all MDs/APPs=triangles.

Symbol color indicates encounter types used in attribution: blue=all medical encounters; orange=evaluation and management encounters only.

Outline indicates years of data used: matching outline=current year only; black outline=lookback to prior year if current year doesn’t result in attribution.

MA = Medicare Advantage; TM=Traditional Medicare; NPI=National Provider Identifier

# Treatment of Advanced Practice Providers as Primary Care Providers

Because advanced practice providers (APPs) are increasingly providing care in specialty settings, particularly cardiology and oncology, and the Centers for Medicare and Medicaid Services (CMS) specialty codes don’t allow us to separate primary care APPs from specialty care APPs, we did not include encounters with APPs in the two primary care provider (PCP) based attribution methods (“PCPs only” and “PCPs then all”). However, other retrospective attribution methods categorize APPs as primary care providers (e.g., the Medicare Shared Savings Program accountable care organization attribution method). To allow comparison of our results with other settings, we show the impact of changing APP treatment in Exhibit S25.

Including APPs as primary care providers in the “PCPs only” method causes a significant increase in the fraction attributed (9-12 percentage points for Traditional Medicare, 6-9 percentage points for Medicare Advantage), however this comes at the cost of similar reductions in stability of attribution. For the “PCPs then all” methods, there is a negligible change in the fraction attributed, but stability decreases by 3-5 percentage points.

## Exhibit S25 – Impact of Including Advanced Practice Providers with Primary Care Physicians


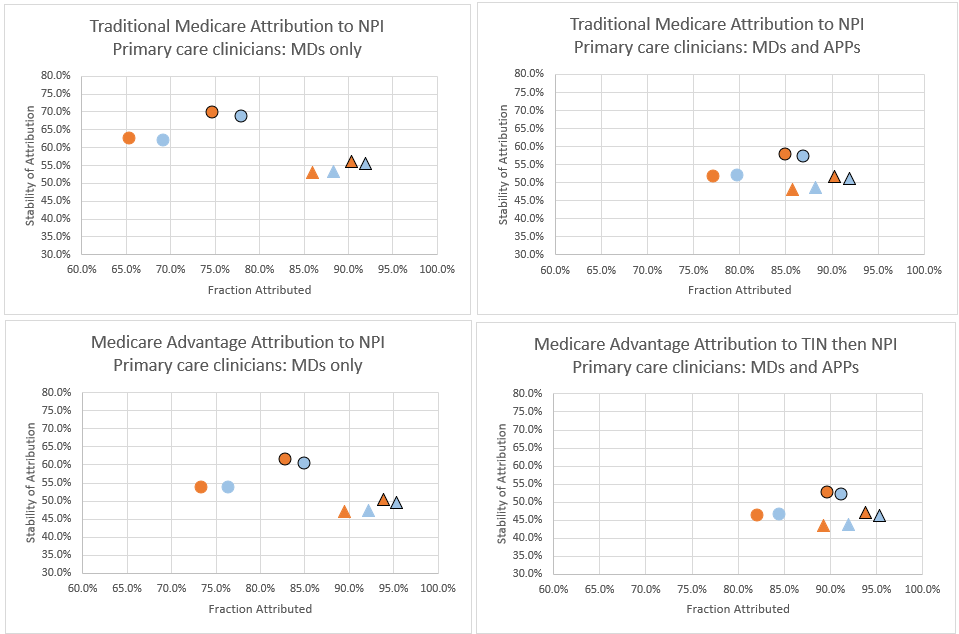


**Legend**

Symbol shape indicates clinician types used in attribution: primary care providers only=circles; primary care providers then all MDs/APPs=triangles.

Symbol color indicates encounter types used in attribution: blue=all medical encounters; orange=evaluation and management encounters only.

Outline indicates years of data used: matching outline=current year only; black outline=lookback to prior year if current year doesn’t result in attribution.

MA = Medicare Advantage; TM=Traditional Medicare; NPI=National Provider Identifier; MD = physician; APP = advanced practice provider

# Attribution Across Health Quartiles

Health risk measures were developed using the Centers for Medicare and Medicaid Services Hierarchical Condition Category (HCC) software.^10^ The software uses claims from the prior year’s inpatient and outpatient encounters to compute the current year’s HCC score. To minimize the impact of differences in MA and TM diagnosis coding intensity,^11^ we follow other work^12^ in excluding diagnoses recorded only on MA chart review records or only in TM or MA encounters for health risk assessment.

The fraction attributed and attribution stability across HCC score quartiles are shown in Exhibit S26 for 2022 attribution using our focal method of attributing based on all medical encounters from primary care providers, then from all physicians and advanced practice providers if the initial attribution isn’t successful. Results are shown separately for Traditional Medicare (TM) and Medicare Advantage (MA) populations. We also show patterns with and without a lookback to the prior year’s data.

When comparing attribution across health risk quartiles, we find both the fraction attributed and attribution stability increase from the first through the third quartile, with very little difference between third and fourth quartile. This suggests that a threshold effect may exist, in which nearly every beneficiary is attributed to a provider once their health risk reaches a certain level. Slopes are slightly higher in TM than MA populations.

## Exhibit S26 – Attribution by Health Risk Quartile


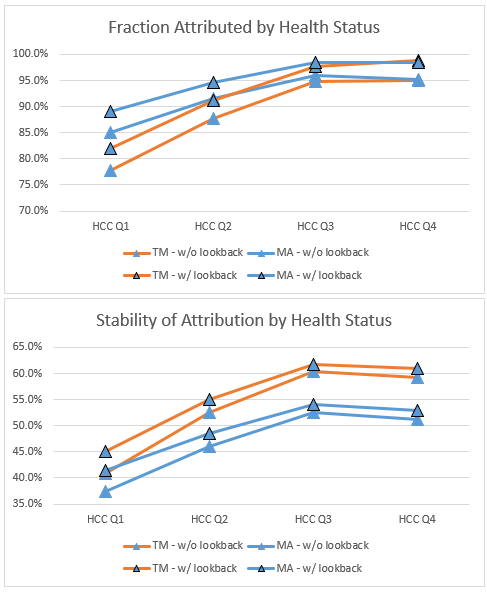


Note: Attribution based on all medical encounters from primary care physicians, then from all medical encounters from physicians and advanced practice providers if initial attribution is unsuccessful.

HCC Q# = Hierarchical Chronic Condition score quartile number #; TM=Traditional Medicare; MA=Medicare Advantage

# Impact of Minimum Provider Information Requirement

We imposed a requirement that at least 50% of a beneficiary’s encounters (medical or evaluation and management, depending on attribution method) had to be mapped to clinician descriptors before we deemed the data of high enough quality that the beneficiary could be attributed. This raises two questions: (1) What is the impact of that minimum? and (2) How would our results change if we increased the minimum requirement for mapped clinician data to 75% or 100% of encounters?

Lack of attribution could arise from several sources: No medical encounters in the year, medical encounters with no clinician descriptors, or no unique plurality even with the tiebreaker. Here we again restrict our analysis to the focal method of attributing based on all medical encounters from primary care physicians, then from all physicians and advanced practice providers if the initial attribution isn’t successful. Using just the current year encounters and our baseline 50% minimum, we see the previously noted higher rates of attribution in the MA population (Exhibit S27), whether we use all medical claims (88.3% TM, 92.1% MA) or E&M claims (85.9% TM, 89.5% MA). This difference is almost completely attributable to the difference in rates of having any eligible encounter recorded. Those enrolled in MA are much more likely to have an interaction with clinicians during the year. This may be a reflection of the higher financial barriers to care in TM, especially when a Medicare Supplement plan is not in place. After our algorithm for enhancing clinician information, very few individuals were unattributed due to the quality of clinician data in either MA or TM.

If we increase our minimum standard for clinician descriptors to at least 75% or 100% of encounters, we find a very small decline in the rates of attribution due to this changing requirement (Exhibit S27). Exhibit S28 provides a picture of how the strictest 100% minimum affects the fraction attributed and attribution stability across all sixteen attribution methods. The largest impacts are 1.0 – 1.5 percentage-point declines in the fraction attributed for four methods relying on medical claims without a lookback. All changes in attribution stability and all other changes in fraction attributed are less than a 1 percentage-point difference.

## Exhibit S27 – Reasons for Lack of Attribution by Minimum Data Requirement

|  | Medical Encounters | | E&M Encounters | |
| --- | --- | --- | --- | --- |
|  | TM | MA | TM | MA |
| 50% minimum clinician information |  |  |  |  |
| Not attributed |  |  |  |  |
| No encounters observed | 10.5% | 6.7% | 12.9% | 9.0% |
| Didn't meet minimum | 0.2% | 0.0% | 0.0% | 0.0% |
| Met minimum but no unique plurality | 1.0% | 1.1% | 1.2% | 1.5% |
| Attributed | 88.3% | 92.1% | 85.9% | 89.5% |
| 75% minimum clinician information |  |  |  |  |
| Not attributed |  |  |  |  |
| No encounters observed | 10.5% | 6.7% | 12.9% | 9.0% |
| Didn't meet minimum | 0.4% | 0.1% | 0.1% | 0.1% |
| Met minimum but no unique plurality | 1.0% | 1.1% | 1.2% | 1.5% |
| Attributed | 88.1% | 92.0% | 85.8% | 89.4% |
| 100% minimum clinician information |  |  |  |  |
| Not attributed |  |  |  |  |
| No encounters observed | 10.5% | 6.7% | 12.9% | 9.0% |
| Didn't meet minimum | 1.5% | 0.4% | 0.3% | 0.3% |
| Met minimum but no unique plurality | 1.0% | 1.1% | 1.2% | 1.5% |
| Attributed | 87.0% | 91.7% | 85.6% | 89.2% |

TM=Traditional Medicare, MA=Medicare Advantage, E&M=evaluation and management

Note: All attributions based on primary care physician encounters, then all physicians and advanced practice providers. Attribution rates shown are prior to lookback adjustments.

## Exhibit S28 – Increasing Minimum Provider Data Requirement from 50% to 100% of Encounters


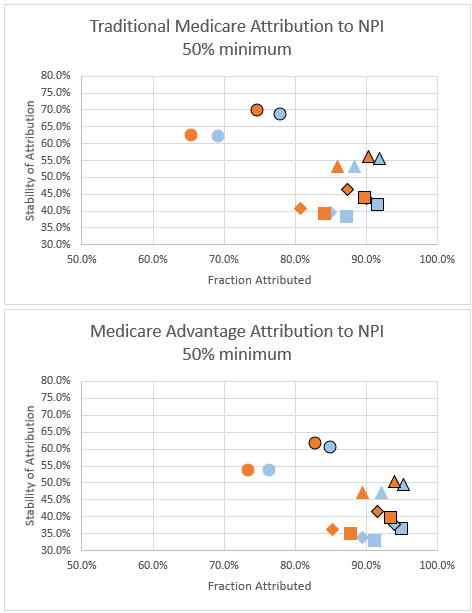

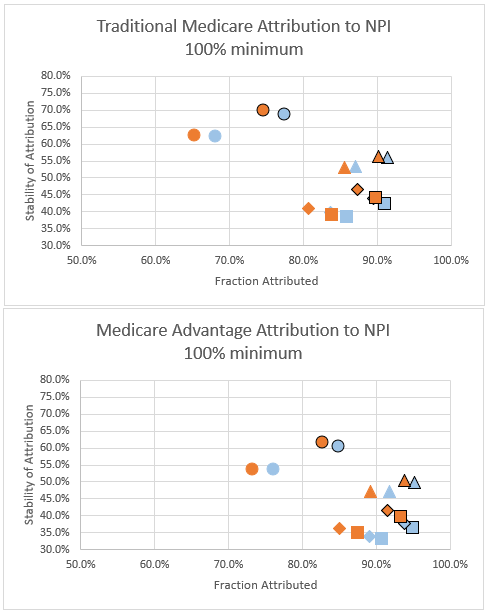


**Legend**

Symbol shape indicates provider types used in attribution: physicians (MDs) and advanced practice providers (APPs)=squares; MDs only=diamonds; primary care providers (PCPs) only=circles; PCPs then all MDs/APPs=triangles.

Symbol color indicates encounter types used in attribution: blue=all medical encounters; orange=evaluation and management encounters only.

Outline indicates years of data used: matching outline=current year only; black outline=lookback to prior year if current year doesn’t result in attribution.

NPI=National Provider Identifier

# Attribution to Specialty Clinicians

Care management or research needs may require the identification of a specialist managing care, rather than identification of a usual source of primary care. In the case of an episode of care involving surgery, such as hip replacement, this may be done by a simple assignment to the lead surgeon. But other conditions, such as cancer treatment, may not involve a triggering event like surgery, and clinician affiliation be less clear cut. Here we identify a population with cancer, using the Chronic Condition Warehouse (CCW) algorithm^13^ to identify individuals with breast, colorectal, endometrial, lung, prostate or urologic cancer, in order to test attribution to oncology specialists.

For this population we use retrospective attribution to identify their primary oncologist. We defined oncology encounters as medical encounters when the provider had a Centers for Medicare and Medicaid Services specialty code of 83, 90, 91, 92 or 98. Attribution was made to the National Provider Identifier (NPI) having a plurality of oncology encounters with the patient, using standardized fees for those encounters as a tiebreaker. Exhibit S29 summarizes the resulting oncology fraction attributed by type of cancer, separately for Traditional Medicare (TM) beneficiaries and Medicare Advantage (MA) enrollees in 2022. We show results with and without the impact of a lookback to the prior year’s data, when the current year did not result in attribution. In general, MA populations had slightly lower fractions attributed relative to TM populations (3-5 percentage points), but the MA slope by cancer type and the impact of lookback was very similar to TM patterns. Lower oncology fraction attributed in MA stands in contrast to the higher rates of general attribution to usual source of care in MA relative to TM populations. In general the fraction attributed is highly sensitive to cancer type, in part because of different intensities of treatment, and variations in the role of primary care in monitoring cancer status. For example, those diagnosed with low-risk prostate cancer are often triaged to “active surveillance,” which involves regular tests and exams that can be performed in primary care, rather than immediate surgery or radiation.^14^ It follows that the oncology fraction attributed would be smaller for this type of cancer than for lung cancer, where more immediate treatment is the norm.

Robustness testing of attribution to tax identification number (TIN) rather than NPI, and of attribution using evaluation and management encounters rather than medical encounters, resulted in nearly identical results. Trends in fraction attributed across calendar years were flat for all cohorts in both coverage types.

## Exhibit S29 – Oncology Attribution


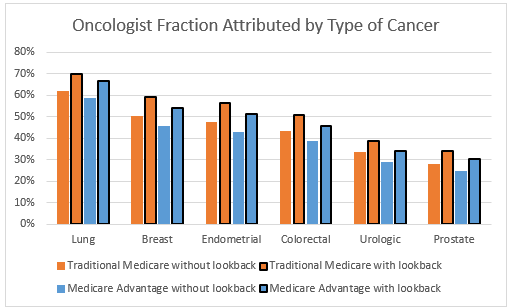


Note: Attribution was assigned based on the plurality of medical encounters with an oncology specialist, using standardized fees for the encounters as a tiebreaker.

# Distribution of Clinicians by Specialty After Data Enhancement

Ultimately, we identify clinician specialty codes for more than 99% of medical encounters in both Traditional Medicare (TM) and Medicare Advantage (MA) data, consistently across years 2016-2022 (manuscript Figure 2, bottom panel). Using the mapping in Exhibit S1, we assign medical claims to the clinician’s credentialling level (primary care physician [PCP], specialty care physician [SCP], advanced practice provider [APP], other). The distribution of medical claims by specialty type is summarized in Exhibit S30. The top panel displays the distribution across all medical claims; the bottom panel across evaluation and management (E&M) claims. These charts show similar trends in MA and TM, documenting a slow shift from physician encounters to encounters with APPs and (for all medical encounters,) other types of providers. Thus, 85% (MA) or 83% (TM) of the medical encounters are mapped to clinician types used in our attribution methodology (SCP, PCP, APP) in 2016, declining to 83% (MA) or 80% (TM) in 2022, with similar trends in MA and TM (Figure 2, top panel). A higher proportion of E&M encounters are mapped to provider types used in attribution. The MA population consistently averages 92-94% of E&M encounters and TM averages 95-96% of E&M claims eligible for attribution algorithms (Figure 2, bottom panel). The remarkable agreement between TM and MA clinician data across the years, including the early years of MA encounter data, suggests that our hierarchical algorithm for determining clinician information creates parity in data quality across coverage types.

## Exhibit S30 – Distribution of Medical Claims by Type of Specialty


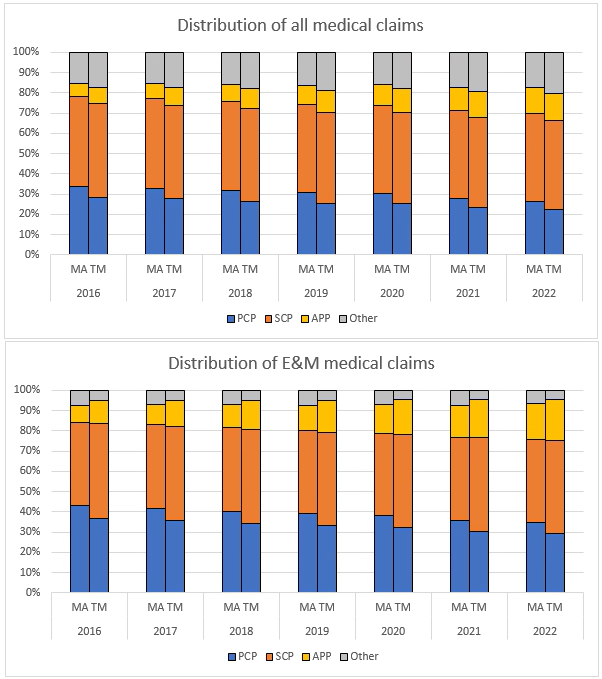


E&M = evaluation and management; PCP = primary care provider; SCP = specialty care provider; APP = advanced practice provider

# Guidelines for Sample SAS Code

Sample SAS code and supporting data files are available for download at <https://github.com/CarolineCarlin/Attribution.git>. The code is available in two formats: as a SAS Enterprise Guide (EG) project and as individual SAS programs. The code assumes the Traditional Medicare (TM) and Medicare Advantage (MA) file structure and variable names match those available in the Centers for Medicare and Medicaid Services (CMS) Virtual Research Data Center (VRDC).^7^

## Data Preparation

The code provided here assumes the TM claims and MA encounter data have been cleaned and formatted using the methods outlined in Jung, Carlin, Feldman and Tran (2022).^6^ The authors originally released sample code for this process through the *Health Services Research* website (<https://onlinelibrary.wiley.com/doi/10.1111/1475-6773.13970>), which has subsequently been updated. As new updates occur, users can assure they are accessing the current code and documentation at <https://github.com/CarolineCarlin/ResourceUse.git>. This data preparation code performs the following activities with CMS VRDC data assumed as the input source:

- Identify MA contracts deemed to have complete submission of encounter data, based on 100% files. For those without access to these full files, indicators of data completeness are included in the data preparation code download.
- Select standard 20% sample, retaining beneficiaries with continuous TM or continuous MA coverage during the year, with full Part A and B coverage. Indicators are created that allow additional filters on the data, including retaining (a) those in the 50 states or District of Columbia (core_state=1), (b) those with full Part D coverage (PtD_full_cvg=1), (c) those without current ESRD status (curr_elig_incl_esrd=0), (d) if MA, those who stay in the same plan (PtC_stay=1), and (e) if MA, those in contracts deemed to submit complete encounter data (MA_complete=1).
- Extract and clean data from the Master Beneficiary Summary File (MBSF) and from Inpatient Facility, Outpatient Facility, Carrier, Durable Medical Equipment, Hospice and Part D Event claims and encounter files. Cleaned MA encounter data are saved in two formats - with and without impact of chart review records included.
- Compute standard fees based on TM claims.
- Append standard fees to TM and MA files to use in measuring resource use.

The attribution code uses the cleaned MBSF, Outpatient Facility and Carrier data.

## Overview of Attribution Code

The sample attribution code process flow (SAS EG) or program directory structure (individual SAS programs) follow the same naming convention. This includes Autoexec programs which load necessary library definitions and formats into memory; Cohort Selection and Data Preparation programs, which extract the relevant encounter data and apply the algorithm to determine clinician information; and three sets of attribution programs that implement three types of attribution algorithms. These three sets of algorithms include Visit-based Attribution with Tiebreaker; Cost-based Attribution with Tiebreaker; and Visit-based Attribution with no Tiebreaker.

All programs include a comment block at the beginning that documents details about the individual program. Each comment block concludes with statements that allow the user to set macro variables that control the program, including the two-digit years that control the span of time for which the user is computing provider attribution.

## Autoexec Programs

These programs should be run to put library name definitions (“Libraries.sas”) and user-defined formats (“Specialty formats.sas”) in memory at the beginning of each SAS session. The Autoexec process flow in the SAS EG version will prompt the user to do so when the project is opened, or the Libraries.sas program can be run with “%include” statement uncommented, to load these definitions in memory at the beginning of a traditional SAS session. The Autoexec folder also contains external data files used in identifying provider specialties. It is assumed that the user has access to the CMS Medicare Data on Provider Practice and Specialty (MD-PPAS) files through the VRDC. The code can be modified to rely on only the included publicly available reference files.

## Cohort Selection and Data Preparation

There are two programs in this section. The first program (“Identify unique encounters and map to specialty.sas”) extracts all medical encounters at the Healthcare Common Procedure Coding System (HCPCS) line level, applying the provider data hierarchical algorithm to determine clinician information for that procedure. The algorithm separates clinicians into primary care physicians (PCPs), specialty care physicians (SCPs), advance practice providers (APPs), and other clinicians. Only medical encounters (HCPCS 90281-99607) are retained, with an indicator identifying evaluation and management (E&M) encounters (HCPCS 99201-99499). Standard fees are captured for use in further attribution work, either as an alternative basis for attribution or as a tiebreaker in the primary visit-based attribution methods.

In the application of the clinician data algorithm, it is assumed the user has access to CMS’s MD-PPAS data file, which provides information about each NPI for whom CMS processed a claim in the year. In addition, it is assumed that the National Plan and Provider Enumeration System (NPPES) and taxonomy mapping reference files included in the download are stored in a directory mapped to the Ext library name.

The second program (“Summarize encounters to NPI-date level.sas”) summarizes from HCPCS level to encounter level, using a three-level summarization to organization’s tax identification number (TIN), provider’s National Provider Identifier (NPI), and encounter date.

## Visit-based Attribution with Tiebreaker

The first two programs perform an attribution directly to the NPI level (“Perform attribution directly to NPI.sas”) and then apply the lookback to enhance the fraction attributed (“Apply lookback to direct NPI attribution.sas”). The initial attribution retains attributed NPI for all beneficiaries in the sample, possibly null if attribution was not achieved, without regard to any requirements about the individual’s minimum rate of provider data quality. If the user wishes to apply the 50% standard used in the manuscript, the variables pct_mapped and pct_EandM_mapped can be used to filter for medical or E&M visit data quality. This minimum standard is included as a macro variable (min_pct) when lookback is applied in the second step, so that the lookback is applied if data quality is poor in the current year. If the user does not have two or more years of data, this second program should not be run as a lookback is not possible.

The next two programs (“Perform attribution to TIN then NPI.sas” and “Apply lookback to TIN then NPI attribution.sas”) perform the same activities, except that attribution is done hierarchically – to the TIN with the plurality of visits, and then within that TIN to the NPI with the plurality of TIN visits. The output file from the first program includes both attributed TIN and NPI, rather than NPI only. When the lookback is applied, the output comprises two files. One file contains the results of lookback at the TIN-NPI level, and the other at the TIN-only level. It is possible that a beneficiary could be attributed to a TIN (so TIN lookback isn’t needed) but have an unbroken tie at the NPI level (so lookback is needed). Thus, the lookback is applied separately for users who ultimately will be using TIN attribution rather than TIN-NPI attribution.

Whether attribution is done directly to the NPI or hierarchically to the TIN-NPI combination, the output files contain one line for each beneficiary, denoting the attributed identifiers for the eight possible attribution methods based on

- medical visits mapped to MDs or APPs
- medical visits mapped to MDs only
- medical visits mapped to MD PCPs only
- medical visits MD PCPs only, supplemented by medical visits to all MDs or APPs if PCP-only attribution fails
- E&M visits mapped to MDs or APPs
- E&M visits mapped to MDs only
- E&M visits mapped to MD PCPs only
- E&M visits MD PCPs only, supplemented by E&M visits to all MDs or APPs if PCP-only attribution fails

Pragmatically, the difference between direct NPI attribution and TIN-NPI attribution is minimal. For the four methods that include PCP-only visits, the agreement between direct and hierarchical NPI attribution is greater than 96% of the beneficiaries. Agreement for the four MD/APP and MD-only methods is less strong, ranging from 88% to 92% of beneficiaries.

## Cost-based Attribution with Tiebreaker

This group of programs parallels the baseline visit-based programs. The only difference is the switch to plurality of standardized fees as the basis of attribution, with plurality of visits as the tiebreaker. There are accompanying changes to the output file names to keep the results distinct.

## Visit-based Attribution with No Tiebreaker

This group of programs parallels the baseline visit-based programs. The only difference is the elimination of plurality of standardized fees as the tiebreaker. There are accompanying changes to the output file names to keep the results distinct.

# References

1. National Bureau of Economic Research. National Plan and Provider Enumeration System Archive. 2024; <https://www.nber.org/research/data/national-plan-and-provider-enumeration-system-nppes>. Accessed February 4, 2025.

2. Centers for Medicare and Medicaid Services. Medicare Claims Processing Manual: Chapter 12 - Physicians/Nonphysician Practitioners. 2024; <https://www.cms.gov/regulations-and-guidance/guidance/manuals/downloads/clm104c12.pdf>. Accessed June 4, 2025.

3. Centers for Medicare and Medicaid Services. Crosswalk: Medicare Provider/Supplier to Healthcare Provider Taxonomy. 2017; <https://www.cms.gov/medicare/provider-enrollment-and-certification/medicareprovidersupenroll/downloads/taxonomycrosswalk.pdf>. Accessed January 28, 2025.

4. Centers for Medicare and Medicaid Services. Medicare Provider and Supplier Taxonomy Crosswalk. 2025; <https://data.cms.gov/provider-characteristics/medicare-provider-supplier-enrollment/medicare-provider-and-supplier-taxonomy-crosswalk/data>. Accessed January 28, 2025.

5. Creighton S, Duddy-Tenbrunsel R, Michel J. The Promise And Pitfalls Of Medicare Advantage Encounter Data. In. *Health Affairs Blog*2019.

6. Jung J, Carlin C, Feldman R, Tran L. Implementation of resource use measures in Medicare Advantage. *Health Serv Res.* 2022;57(4):957-962.

7. Chronic Conditions Data Warehouse. About the VRDC and Requesting Access. n.d.; <https://www2.ccwdata.org/web/guest/about-vrdc>. Accessed June 4, 2025.

8. Ganguli I, Souza J, McWilliams JM, Mehrotra A. Practices Caring For The Underserved Are Less Likely To Adopt Medicare's Annual Wellness Visit. *Health affairs.* 2018;37(2):283-291.

9. MedPAC. Medicare and the Health Care Delivery System. *MedPAC Report to Congress* 2019; June 2019:<https://www.medpac.gov/wp-content/uploads/import_data/scrape_files/docs/default-source/reports/jun19_ch7_medpac_reporttocongress_sec.pdf>.

10. Centers for Medicare and Medicaid Services. Risk Adjustment. n.d.; <https://www.cms.gov/Medicare/Health-Plans/MedicareAdvtgSpecRateStats/Risk-Adjustors>. Accessed 05/31/2022.

11. MedPAC. Medicare Payment Policy. *MedPAC Report to Congress* 2022; March 2022:<https://www.medpac.gov/wp-content/uploads/2022/03/Mar22_MedPAC_ReportToCongress_SEC.pdf>. Accessed 09/18/2022.

12. Jung J, Carlin C, Feldman R. Medicare Advantage Has Lower Resource Use and Better Quality of Care than Traditional Medicare. *American Journal of Health Economics.* 2024;Forthcoming.

13. Centers for Medicare and Medicaid Services. 30 CCW Chronic Conditions Algorithms. 2023; <https://www2.ccwdata.org/documents/10280/19139421/chr-chronic-condition-algorithms.pdf>. Accessed 03/15/2023.

14. American Cancer Society. Observation or Active Surveillance for Prostate Cancer. n.d.; <https://www.cancer.org/cancer/types/prostate-cancer/treating/watchful-waiting.html>. Accessed June 18, 2025.
